# Cathepsin H Knockdown Reverses Radioresistance of Hepatocellular Carcinoma via Metabolic Switch Followed by Apoptosis

**DOI:** 10.3390/ijms24065257

**Published:** 2023-03-09

**Authors:** Qiao Chen, Shugen Qu, Zhenzhen Liang, Yi Liu, Huajian Chen, Shumei Ma, Xiaodong Liu

**Affiliations:** 1 Department of Radiation Medicine, School of Public Health, Jilin University, Changchun 130021, China; 2 Department of Radiation Medicine, School of Public Health and Management, Wenzhou Medical University, Wenzhou 325035, China

**Keywords:** hepatocellular carcinoma, radioresistance, glycolysis, aerobic respiration, apoptosis, radiotherapy

## Abstract

Despite the wide application of radiotherapy in HCC, radiotherapy efficacy is sometimes limited due to radioresistance. Although radioresistance is reported with high glycolysis, the underlying mechanism between radioresistance and cancer metabolism, as well as the role of cathepsin H (CTSH) within it, remain unclear. In this study, tumor-bearing models and HCC cell lines were used to observe the effect of CTSH on radioresistance. Proteome mass spectrometry, followed by enrichment analysis, were used to investigate the cascades and targets regulated by CTSH. Technologies such as immunofluorescence co-localization flow cytometry and Western blot were used for further detection and verification. Through these methods, we originally found CTSH knockdown (KD) perturbed aerobic glycolysis and enhanced aerobic respiration, and thus promoted apoptosis through up-regulation and the release of proapoptotic factors such as AIFM1, HTRA2, and DIABLO, consequently reducing radioresistance. We also found that CTSH, together with its regulatory targets (such as PFKL, HK2, LDH, and AIFM1), was correlated with tumorigenesis and poor prognosis. In summary, our study found that the cancer metabolic switch and apoptosis were regulated by CTSH signaling, leading to the occurrence of radioresistance in HCC cells and suggesting the potential value of HCC diagnosis and therapy.

## 1. Introduction

Hepatocellular carcinoma (HCC) is one of the most common malignancies with a poor prognosis. Radiotherapy (RT) is widely applied in HCC patients, but its efficacy is limited due to inherent or acquired radioresistance [1,2,3]. Recently, evidence has shown that the cancer metabolic switch from aerobic respiration to glycolysis (with predominance in the Warburg effect) promotes radioresistance in several cancer types [4,5,6,7,8,9,10]. However, how the cancer metabolic switch regulates radiation reactivity in liver cancer remains unclear.

Aberrant metabolism is a major hallmark of cancer [11]. In order to gain a survival advantage, cancer cells flexibly develop abnormal metabolic networks through different pathways [12]; a core component is the Warburg effect (aerobic glycolysis as the predominance). Although glycolysis is less efficient at producing ATP than aerobic respiration, intermediate glucose metabolism between aerobic respiration and aerobic glycolysis can shunt anabolic pathways such as lipid synthesis (adipogenesis), amino acid production, and nucleotide synthesis, thereby promoting tumor cells’ sustainable proliferation and progression and avoiding the occurrence of apoptosis [13,14]. Moreover, high-level aerobic glycolytic metabolism also helps cells avoid massive reactive oxygen species (ROS) accumulation without compromising the cancer cells’ energy demand and thus keeping them away from overloaded oxidative stress, which is another main cause of apoptosis [14,15,16]. Therefore, glycolysis is often used as the metabolic basis in tumors. Although several studies have reported radioresistance with high rates of glycolysis [17,18,19], the exact metabolic mechanism of ionizing radiation (IR) responsiveness remains unclear.

IR triggers various forms of cell death, including apoptosis. After IR treatment, second messengers and damaged DNA can mediate apoptosis [20,21]. However, how cancer metabolites are involved in IR-induced apoptosis remains elusive.

The family of cathepsins is involved in overall protein turnover and specific cellular processes, such as apoptosis, antigen presentation, and prohormone processing [22,23]. Despite being implicated in cancer development [24] and the processing of neurotransmitters [25], the role of CTSH, a ubiquitously expressed lysosomal cysteine protease in cancer apoptotic process, is rarely reported [26,27,28].

To solve the elusive questions above, here, we outline a comprehensive CTSH-involved metabolic mechanism to regulate apoptosis and radioresistance, suggesting novel therapeutic strategies in radiation sensitization via disrupting cancer metabolism.

## 2. Results

### 2.1. Radiation-Inhibited Tumor Growth and Induced Apoptosis In Vivo

**Figure 1:**
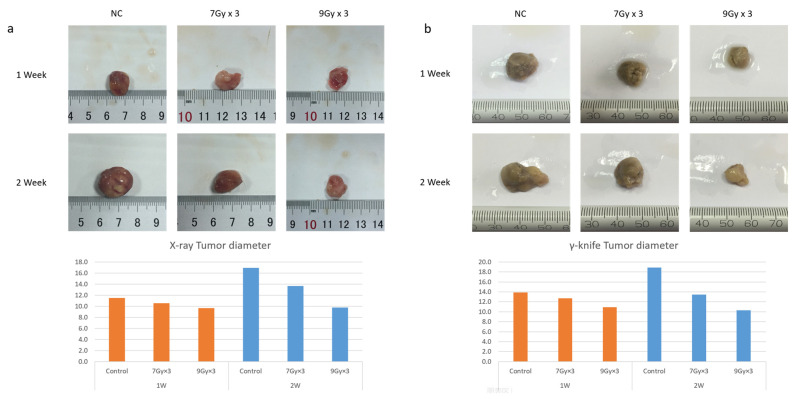

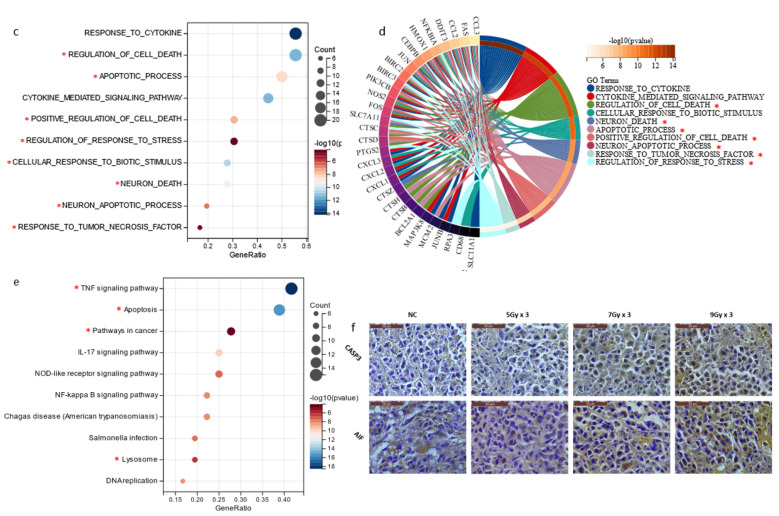
Radiation Inhibited Tumor Growth and Induced Apoptosis in vivo. (**a**,**b**) Tumor-bearing rat models were established. The IR group was given 7Gy × 3 and 9Gy × 3 fractions imitating SBRT using an X-ray accelerator (**a**) and gamma-knife (**b**), respectively, and the tumors were harvested and measured 7/14 days after IR treatment; (**c**,**d**) GO biological process (BP) enrichment analysis of the selected top up-regulated gene set—the terms we were interested in are marked with *; (**e**) KEGG enrichment analysis of the top up-regulated gene set—the terms we were interested in are marked with *; (**f**) Immunohistochemical staining of tumor tissues after different doses of IR, Caspase3, and AIF antibodies.

First, IR treatment was applied to rats bearing an orthotopic tumor in the liver. Compared to the control group, tumor growth was significantly inhibited in IR treatment groups, and this effect was more obvious when the dose was added to 9 Gy × 3 fraction (Figure 1a,b; Supplementary Figure S1). Then, to further detect the mechanism of tumor inhibition induced by IR, transcriptome sequencing was conducted. The most significantly increased genes were collected. Biological process (BP) enrichment analysis showed that the up-regulated genes after IR were highly related with cell death, especially apoptosis (Figure 1c,d). In addition, with KEGG enrichment analysis, apoptosis and the TNF pathway seemed to be closely related to these genes (Figure 1e). Consistently, immunohistochemical (IHC) staining showed positive results of Caspase3 and the apoptosis-inducing factor (AIF) after IR, which confirmed the occurrence of apoptosis (Figure 1f).

### 2.2. CTSH Participated in Radioresistance Regulation of HCC Cells

**Figure 2:**
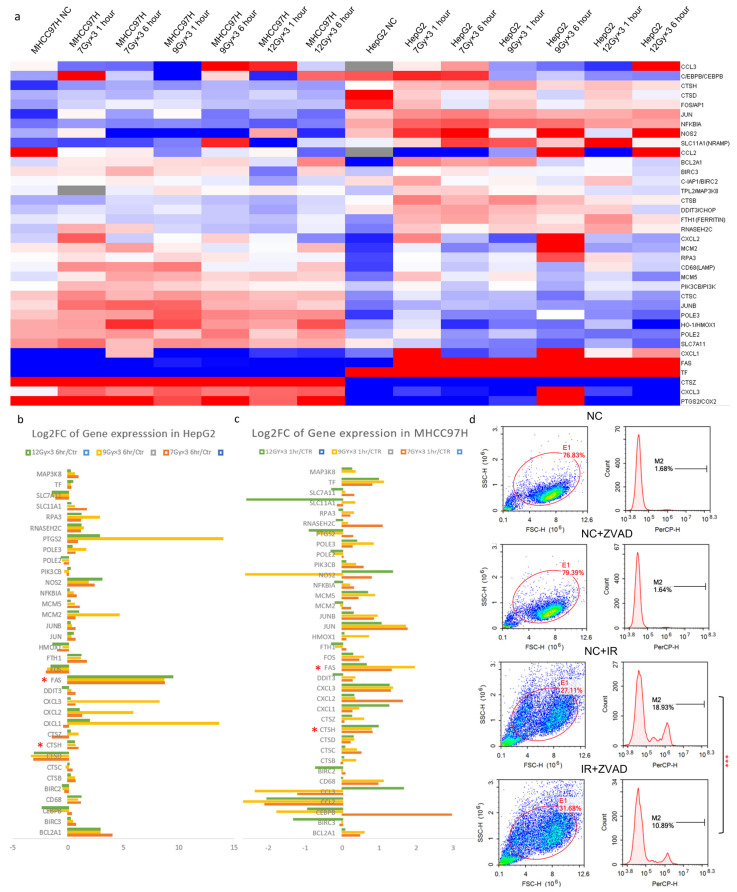

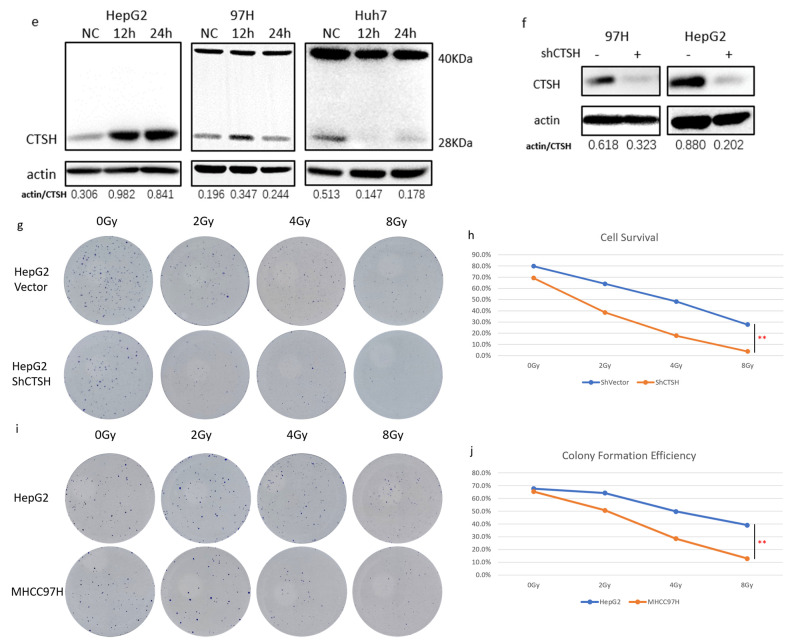
CTSH Participated in Radioresistance Regulation of HCC Cells. (**a**) Heatmap of in vitro PCR array on involved genes from in vivo sequencing; HepG2 and MHCC97H cell lines were used for different doses, and then cells were examined 1 h and 6 h after radiation (IR); (**b**,**c**) Histograms of gene expression before and after IR in HepG2 and MHCC97-H cell lines; (**d**) Flow cytometry after trypan blue staining verified cell death after IR (*p* < 0.05). ZVAD (inhibitor of apoptosis) was applied to cells before IR treatment; (**e**) Western blot result of CTSH protein expression in HepG2, MHCC97-H, and Huh7 cells before and after IR; (**f**) Western blot analysis of CTSH expression for the verification of CTSH knockdown; (**g**,**h**) Colony formation assay to compare radio-sensitivity between CTSH knockdown and vector cells (*p* < 0.05); (**i**,**j**) Colony formation assay to compare radio-sensitivity between wildtype HepG2 and MHCC97-H cells (*p* < 0.05). * *p* < 0.1; ** *p* < 0.05; *** *p* < 0.01.

Due to the non-homology between human and rat models [29], to further confirm the change of genes in HCC cells, a PCR array was performed on MHCC97H and HepG2 cell lines focusing on collected genes from in vivo experiment (Figure 2a–c, Attachment File 1). As transcription always happens earlier than translation, we examined the cell lines at 1 and 6 h after IR, and a PCR array was conducted expression profiles were established. The genes expressed consistently with in vivo sequencing results were found (Figure 2b,c). Meanwhile, trypan blue staining flow cytometry verified cell death after IR. Inhibitors of apoptosis, ferroptosis, necrosis, and autophagy pretreatment were applied to HCC cells, and IR-induced cell death was found to be reversed by the pretreatment of ZVAD (commonly used as an apoptosis inhibitor). With the pretreatment of ZVAD, the value of the MHCC97H cell death signal peak was significantly reduced after IR (Figure 2d right), while in other groups, the rescue effect was not evident (Supplementary Figure S2A). Previous results had already informed us of the involved genes in vivo (Figure 1c–e). After filtering out genes expressed inconsistently with the in vivo results or those already well-reported [22,30,31,32], we mainly focused on CTSD, CTSH and FAS (marked with * in Figure 2b,c; Supplementary Figure S2B). Then, due to the low abundance of FAS and the inconsistency of CTSD expression (Figure 2e; Supplementary Figure S2C), we abandoned FAS, CTSD and selected CTSH for further study. 

To further understand the influence of CTSH, a CTSH knockdown model was established in HepG2 cells (Figure 2f), and the radio-sensitivity was examined using a colony formation assay. The results showed that knockdown (KD) of CTSH significantly increased the radio-sensitivity of HepG2 cells (Figure 2g,h). Consistently, MHCC97H cells showed significantly higher radio-sensitivity compared with HepG2 (Figure 2i,j), with a lower CTSH expression after IR stimulation (Figure 2e). Taking these results together, we realized that CTSH participated in the maintenance of radioresistance in HCC cells.

### 2.3. Restrained Glycolysis and Promoted Aerobic Respiration Inhibited Radioresistance of HepG2 Cells

**Figure 3:**
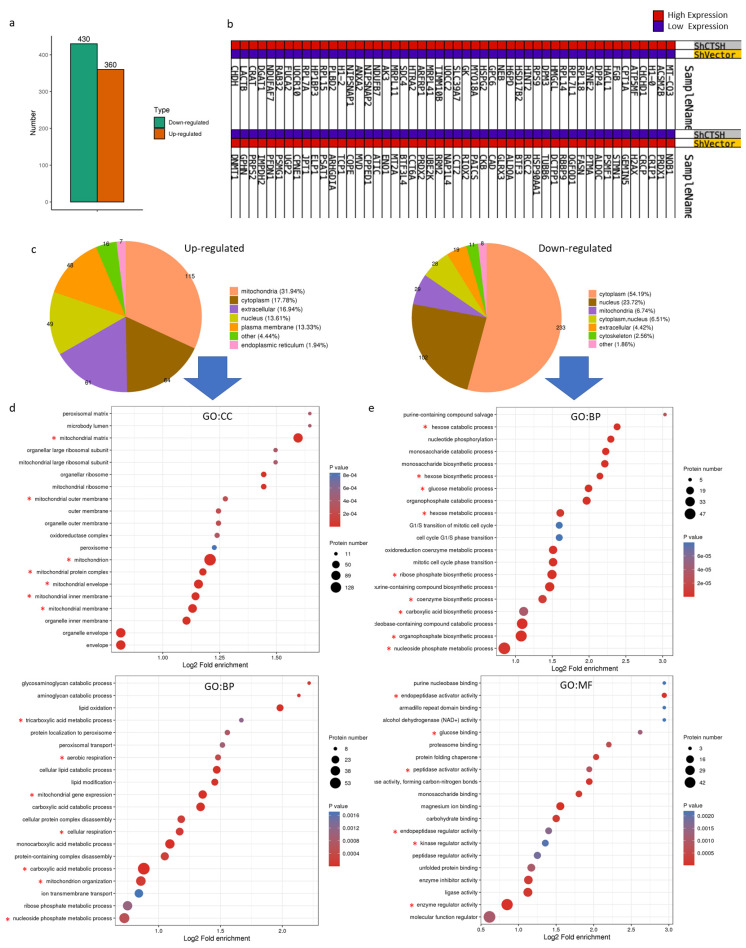

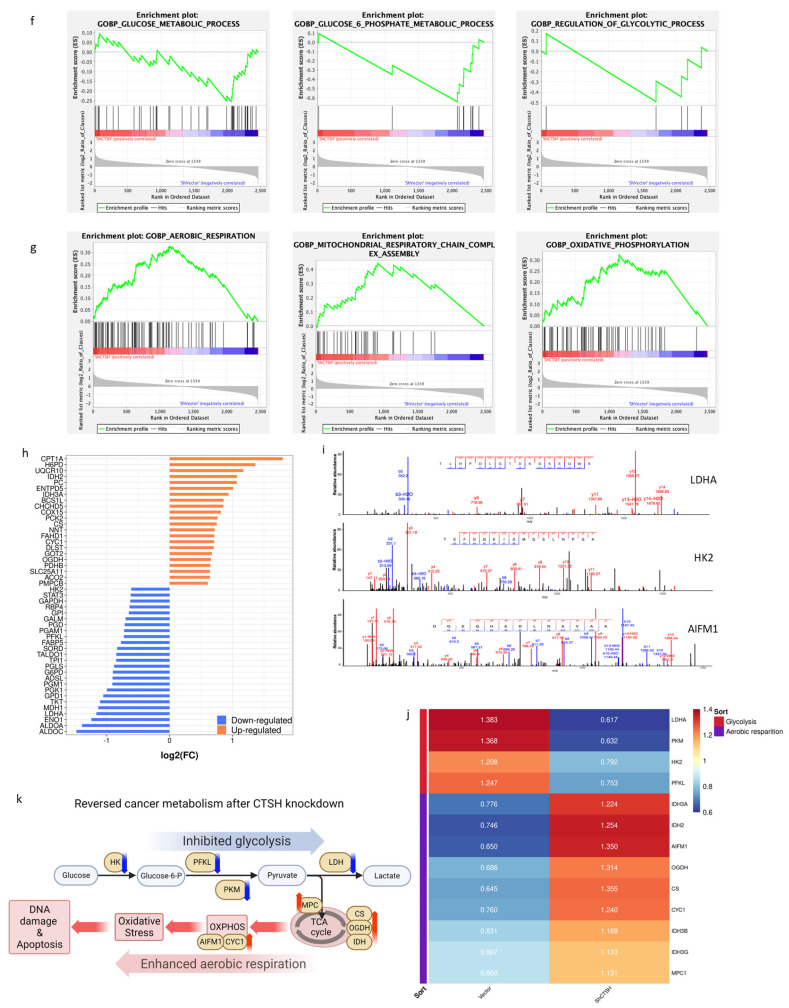
Restrained Glycolysis and Promoted Aerobic Respiration Inhibited Radio-resistance of HepG2 Cells. (**a**) The proteome mass spectrometry on CTSH knockdown/Vector cells compared with Vector cells. A total of 430 genes were down-regulated and 360 were up-regulated after CTSH knockdown; (**b**) Heatmap of the top 50 up-regulated and down-regulated genes in subfigure (**a**); (**c**) Pie chart to show constituents among the up-regulated and down-regulated genes after CTSH knockdown; (**d**) The gene ontology (GO) cell component (CC) and biological process(BP) enrichment analysis of increased proteins after CTSH knockdown. The terms we were interested in were marked with *; (**e**) The GO:BP and molecular function (MF) enrichment analysis of decreased proteins after CTSH knockdown; (**f**) GSEA results to show the inhibition of glucose metabolic process (*p* < 0.05) and regulation of glycolytic biological process (*p* < 0.1) after CTSH knockdown; (**g**) GSEA results to show the up-regulation of aerobic respiration (*p* < 0.05), mitochondrial respiration chain (*p* < 0.05), and oxidative phosphorylation biological process (*p* < 0.05) after CTSH knockdown; (**h**) Histograms of the top 25 changing genes related to glucose metabolism after CTSH knockdown; (**i**) LDHA and HK2 (and other proteins) specifically detected by the probe of mass spectrometry; (**j**) Protein level changes of selected glucose-metabolism-related genes after CTSH knockdown (by mass spectrometry); (**k**) Graphical abstract of this part and the corresponding protein change.

To further understand how CTSH performs in regulating HCC metabolism and radiation resistance, proteome mass spectrometry (MS) was performed. As the MS results showed, 360 proteins were up-regulated and 430 were down-regulated after CTSH knockdown (Figure 3a,b). Further enrichment analysis showed the up-regulated proteins mainly localized in the mitochondria (Figure 3c). In addition, these up-regulated proteins were related to mitochondrial function and aerobic respiration, while the down-regulated ones were associated with biosynthesis and metabolic processes (Figure 3d,e). As mitochondria are known to play an important role in metabolism and apoptosis [33] and the cancer metabolic switch is important for cancer cell fate [34], we wondered whether CTSH was affecting cell fate by regulating the metabolic switch. Remarkably, more detailed GSEA results confirmed our hypothesis; after CTSH knockdown, the glycolytic metabolism was inhibited, while the aerobic respiration was promoted (Figure 3f,g). Important molecules for glycolysis such as HK2, PFKL, PKM, and LDHA were all down-regulated. Furthermore, key factors of the tricarboxylic acid cycle (TCA), such as CS, OGDH, and IDH, and oxidative phosphorylation (OXPHOS)-promoting factors, such as AIFM1 and CYC1, were up-regulated (Figure 3h–j). At the same time, mitochondrion pyruvate carrier MPC1 increased, indicating an increase in mitochondria pyruvate intake. Thus, enhanced TCA and OXPHOS formed a strong up-regulation of the aerobic respiration cascade (Figure 3h–j). Considering the reported association of glycolysis with radioresistance, the above results indicate that CTSH knockdown inhibited the radioresistance of HepG2 cells by perturbing glycolysis and reversing the cancer metabolism to aerobic respiration (Figure 3k).

### 2.4. Knockdown of CTSH and Enhanced Aerobic Respiration Promoted Radiation-Induced Apoptosis via IAP Inhibition and AIF Signal

**Figure 4:**
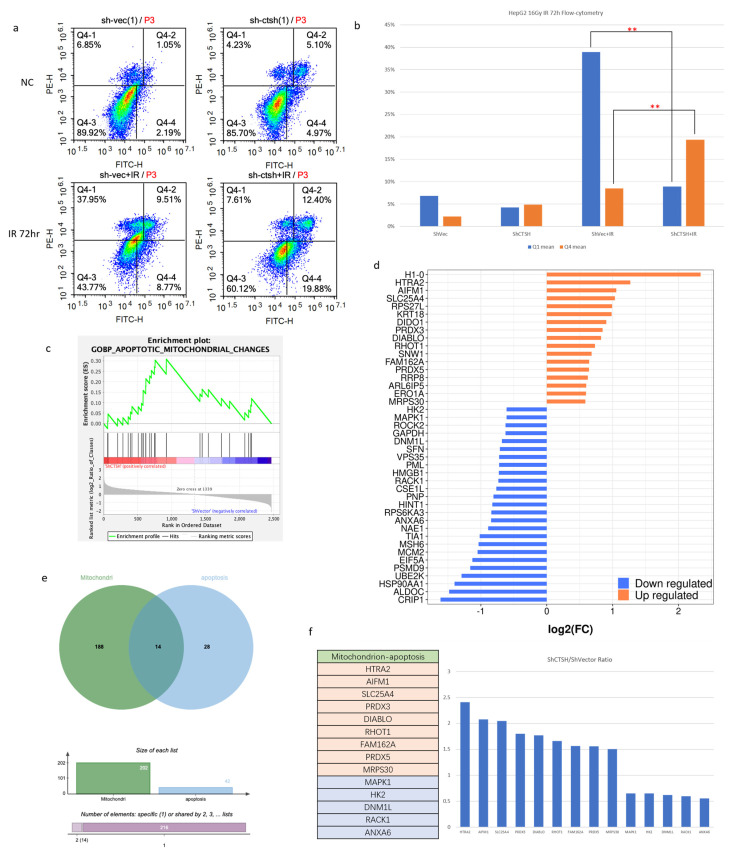

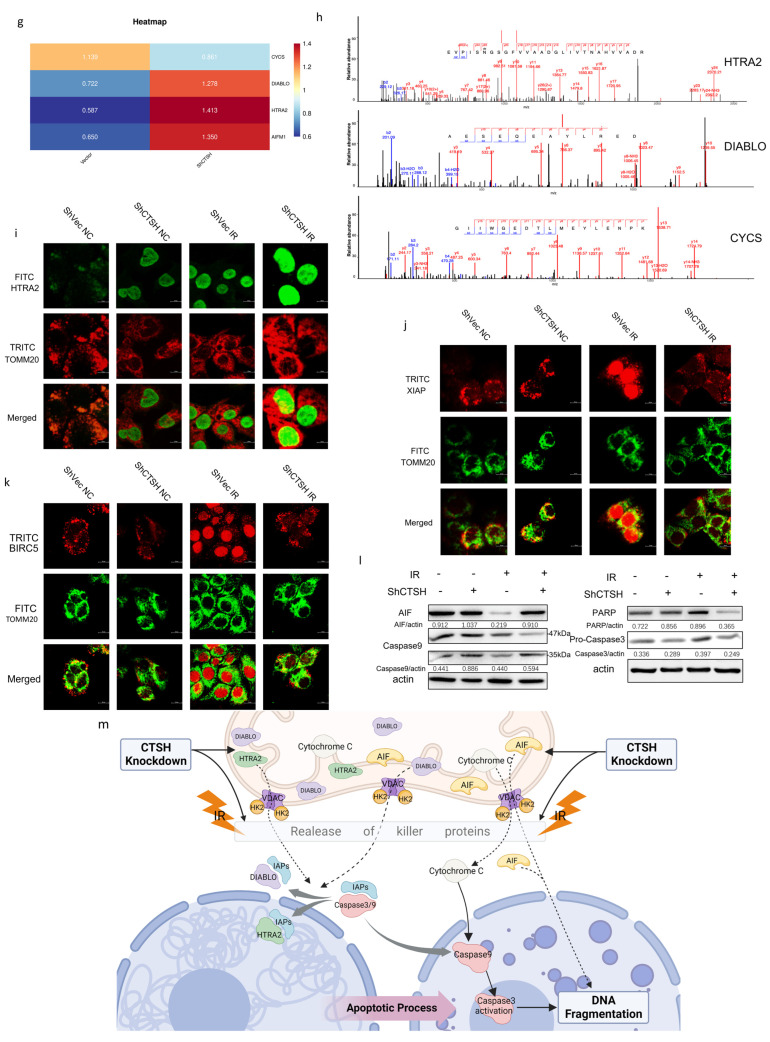
Knockdown of CTSH and Enhanced Aerobic Respiration Promoted Radiation-induced Apoptosis via IAP Inhibition and AIF Signaling. (**a**,**b**) Flow cytometry analysis of Annexin V/PI staining of shCTSH/vector cells. The IR groups were given a 15Gy dose and harvested after 72 h (*p* < 0.05); (**c**) GSEA results to show the biological process change of apoptotic mitochondrial change after CTSH knockdown (*p* < 0.05); (**d**) Histogram showing the changes of apoptosis-related genes in protein level; (**e**) intersection of apoptosis and mitochondrial-related genes. (**f**) Among the intersection, 14 genes, including 9 up−regulated and 5 down-regulated, were collected; (**g**) Changes in protein level of focused and selected glucose-metabolism-related genes after CTSH knockdown detected by mass spectrometry; (**h**) HTRA2, DIABLO, and CYCS (and other proteins) were specifically detected by the probe of mass spectrometry; (**i**) HTRA2 activity (FITC fluorescence) in shCTSH/vector HepG2 cells with/without IR was detected by an immunofluorescence co-localization assay. TOMM20 was used to track the mitochondria with TRITC fluorescence (1 μm scale bar added); (**j**,**k**) XIAP and BIRC5 activities (TRITC fluorescence) in shCTSH/vector HepG2 cells with/without IR were detected by an immunofluorescence co-localization assay. TOMM20 was used to track the mitochondria with FITC fluorescence (1 μm scale bar added); (**l**) Apoptotic phenotype expression of AIF, PARP, Pro−Caspase3, and Caspase9 were detected by Western blot; (**m**) Graphical abstract of this part of the results. ** *p* < 0.05.

As the involvement of CTSH in apoptosis was found by our bioinformatic analysis (Figure 1d,e), to directly confirm this, an Annexin V−PI staining flow cytometry was performed. The results showed that apoptosis was significantly promoted by CTSH knockdown in both IR and NC conditions (Figure 4a,b). Referring to our previous results (Figure 2d) and the up-regulation of AIFM1 (an apoptosis-inducing factor) (Figure 3j), to determine which signaling was involved during the proapoptotic process, we analyzed the MS results using GSEA. The results suggested a promotion of apoptosis; among these, many apoptotic-related genes were affected after CTSH knockdown (Figure 4c,d). Thus, to determine the target of CTSH apoptotic regulation through mitochondrial signaling, an intersection of apoptosis and mitochondrial-related genes was taken; a total of 14 genes, including 9 up-regulated and 5 down-regulated, were listed (Figure 4e,f). Among them, HTRA2 and DIABLO were up-regulated (Figure 4g,h), and they were both reported to promote the apoptotic process as inhibitors of apoptosis (IAPs) [35,36,37]. An immunofluorescence co-localization assay further verified our findings; compared to the blank-load transfection group (ShVec) and the control group (NC), HTRA2 and DIABLO increased in the CTSH knockdown group and inhibited the activity of the IAPs (XIAP and Survivin) in irradiated CTSH knockdown cells, and the inhibition of the IAPs occurred within both the cell nucleus and the cytoplasm (Figure 4i–k; Supplementary Figure S3). Accumulating data support that tumor suppression may be achieved by inhibiting glycolysis and promoting OXPHOS (part of the aerobic respiratory chain) [35]. As a killer protein, besides promoting OXPHOS, the up-regulated AIFM1 (Figure 4g) is also known to induce DNA fragmentation during the apoptotic process [38,39,40,41], suggesting a proapoptotic effect based on the up-regulated aerobic respiration cascade. Furthermore, the increased (activated) caspase family (Caspase9 and Caspase3 cleavage) expression finally confirmed these connections (Figure 4l). From these results, we realized that the proapoptotic effect of CTSH knockdown is carried out through promoted IAP inhibition and enhanced aerobic respiration, and this process is executed by HTRA2, DIABLO, and AIF signaling (Figure 4m).

### 2.5. CTSH Knockdown Changed Mitochondrial Membrane Permeability and Stability in Proapoptotic Signaling

**Figure 5:**
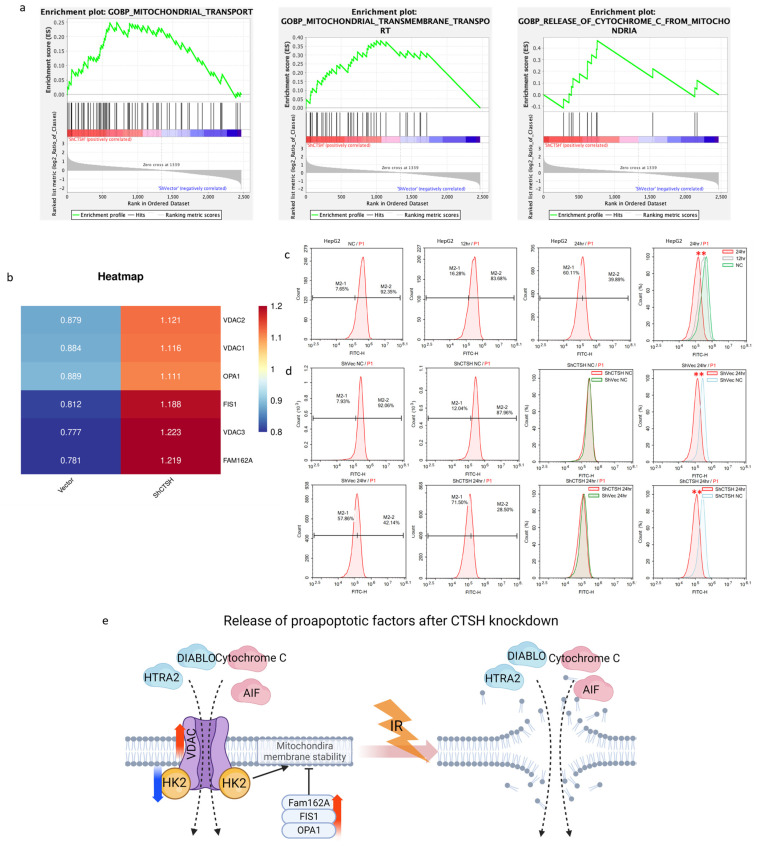
CTSH Knockdown Changed Mitochondrial Membrane Permeability and Stability in Proapoptotic Signaling. (**a**) GSEA results showing the up-regulation of the transmembrane transport of mitochondria and the release of cytochrome c biological processes (BPs) after CTSH knockdown (*p* < 0.05); (**b**) Increase in the VDAC family, Fam162A, FIS1, and OPA1 in protein level after CTSH knockdown detected by mass spectrometry; (**c**) Mitochondrial membrane potential (MMP) of wildtype HepG2 cells with and without IR treatment detected by flow cytometry (*p* < 0.05); (**d**) Mitochondrial membrane potential of CTSH vector/knockdown HepG2 cells with and without IR treatment detected by flow cytometry (*p* < 0.05); (**e**) Graphical abstract of the above findings and the corresponding changes in protein level. ** *p* < 0.05.

Apoptosis includes intrinsic (mitochondrial) and extrinsic (death receptor) mechanisms. As previous results had suggested the involvement of mitochondrial genes (Figure 3d,e and Figure 4c,d), we decided to focus on mitochondrial dysfunction and apoptosis for further study. Remarkably, the GSEA results showed an increased cascade of the transmembrane transport of mitochondria and cytochrome c release (Figure 5a). Among those, VDAC formed a channel through the mitochondrial outer membrane and allowed the diffusion of hydrophilic molecules. It opened at low or zero membrane potential and closed at potentials above 30–40 mV [42]. Fam162A, FIS1, and OPA1 were reported to be involved in proapoptotic factor release (such as cytochrome c), caspase activation (such as CASP9), and mitochondrial permeability transition induction [43,44,45]. Interestingly, after CTSH knockdown, all these genes were up-regulated (Figure 5b). Besides glycolysis, HK2 also plays a role in maintaining the integrity of the outer mitochondrial membrane and preventing the release of apoptogenic molecules from the intermembrane space and subsequent apoptosis [33]. In cancer cells, it binds to and inhibits VDAC to suppress mitochondrial function while stimulating glycolysis [46], and it has also been observed to be down-regulated (Figure 3j). All these results indicated an increase in the permeability and instability of the mitochondrial membrane. Mitochondrial membrane potential (MMP) examination after IR further confirmed the participation of these molecules (Figure 5c,d); a decrease in MMP (IR-induced) and membrane stability (CTSH KD−induced) made it easier for molecules to be released from the mitochondria after IR, thus promoting the apoptotic process. The above results suggested an explanation responsible for the increased apoptotic flux; CTSH knockdown promoted the release of some proapoptotic factors after IR through modulating the permeability and stability of the mitochondrial membrane (Figure 5e).

### 2.6. CTSH and Targets Were Correlated with Tumorigenesis and Poor Prognosis

**Figure 6:**
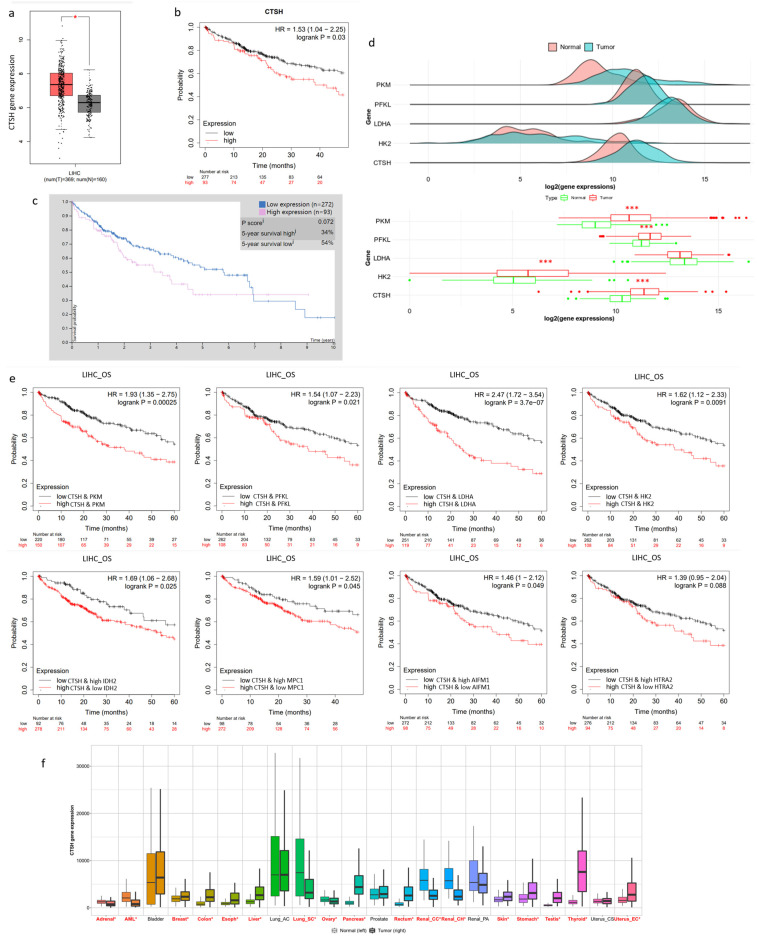

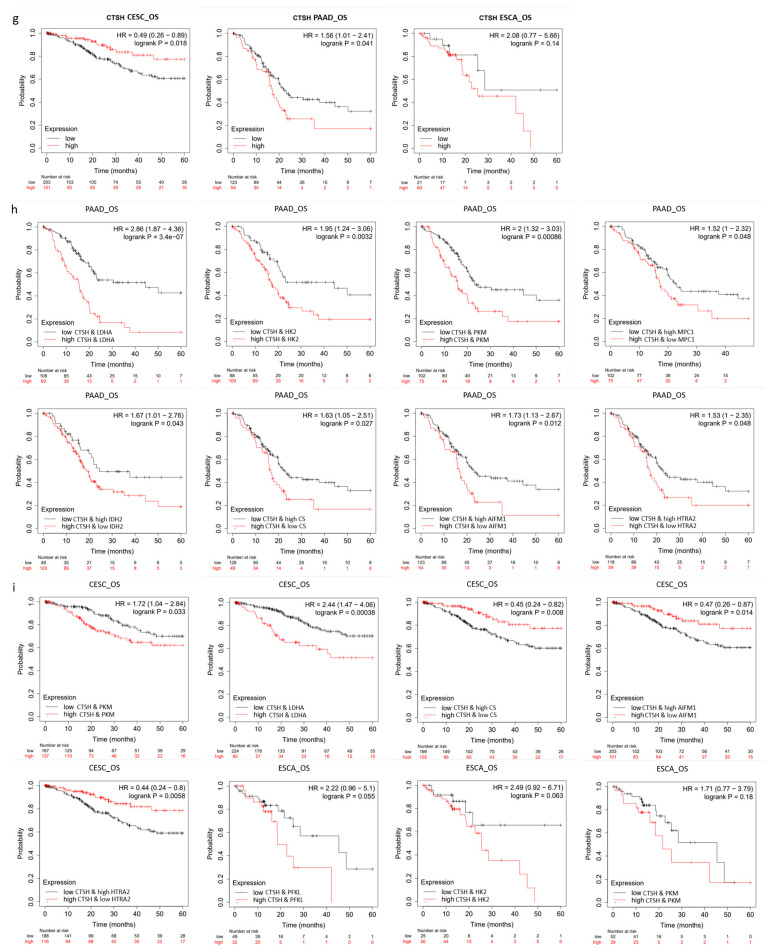
CTSH and Its Targets Were Correlated with Tumorigenesis and Poor Prognosis. (**a**) Expression of CTSH gene in HCC tumor and normal tissues by GEPIA; (**b**) Kaplan–Meier survival plot of CTSH gene expression of HCC by Kaplan–Meier Plotter; (**c**) Survival plot of CTSH protein expression between high and low level analyzed by The Human Protein Atlas; (**d**) Multi-gene expression analysis of CTSH with its downstream targets by TNMplot; (**e**) Survival plot of CTSH gene combined with its targets’ expression in HCC; (**f**) Expression profile of CTSH between tumor and normal tissues in human cancers; (**g**) Survival plot of CTSH gene in cervical squamous-cell carcinoma (CESC), pancreatic adenocarcinoma (PAAD), and esophageal squamous-cell carcinoma (ESCA); (**h**,**i**) Survival plot of CTSH combined with its targets’ expression in the above cancers; * *p* < 0.1; *** *p* < 0.01.

Then, to verify the potential of CTSH in clinical application, a series of bioinformatic analyses were performed using well-known databases. For the mRNA level, CTSH showed significantly higher expression in HCC than non-tumor samples (Figure 6a) (derived from GEPIA). In addition, in a clinical cohort of 370 HCC patients, using a validated mRNA signature, higher CTSH signaling was associated with poor prognosis (*p*-value = 0.025) (Figure 6b) (derived from Kaplan–Meier plotter). A survival Kaplan–Meier (KM) analysis of 365 patients showed similar results in the CTSH protein level (Figure 6c) (from The Human Protein Atlas). A multi-gene expression comparison of CTSH targets, especially those glycolysis-related genes, showed homogeneous trends related to tumorigenesis (Figure 6d) (from TNMplot). Furthermore, by combining CTSH expression and its downstream targets expressions (i.e., CTSH combined with PFKL, PKM, LDHA, HK2; and low IDH, MPC1, AIFM1, and HTRA2 combined with high CTSH expression), we found a remarkably significant facilitation of tumorigenesis and poor prognosis in HCC (Figure 6e) (Kaplan–Meier plotter). Then, to verify the universality of the above findings, profiles of CTSH expression were drawn out using two different databases (Figure 6f and Figure S4A) (TNMplot and GEPIA). Among them, human malignancies were selected because of their high CTSH tumor expression. Remarkably, CTSH was related with poor prognosis in all these cancers (Figure 6g). Furthermore, survival KM plotting showed consistent facilitations of poor prognosis in malignancies such as cervical squamous-cell carcinoma, esophageal carcinoma, and pancreatic adenocarcinoma (Figure 6h,i and Figure S4B,C) (Kaplan–Meier plotter). However, in other low-grade malignancies, this relationship seems insignificant or even reversed (Supplementary Figure S4D). Taken together, these findings implied significant therapeutic and diagnostic potential for clinical use.

## 3. Discussion

RT is increasingly used in advanced HCC and has been reported to confer survival benefits [1,2,3]. Nevertheless, radioresistance has been a major hurdle. Several mechanisms of radioresistance encompassing different molecular pathways in HCC have been suggested [47,48,49,50,51], but most of the previous studies have mainly addressed one single signaling in mediating resistance and have not illustrated a general mechanism shared by different resistant backgrounds. To solve these problems, in the current study, we first attempted to characterize the radio-sensitivities of different HCC cells by attributing them to the differing CTSH-mediated metabolic styles, with the aim of better delineating the mechanism underlying radioresistance encountered in clinical practice.

### 3.1. CTSH-Modulated Metabolic Switch from Aerobic Respiration to Glycolysis Is Important for Radioresistance of HCC

It has recently been reported that the cancer metabolic switch plays a role in resistance after treatment, such as through highly activated glycolysis, enhanced lipogenesis or fatty acid beta-oxidation, and increased nucleotide metabolism [4,5,6,7,8,9,10,47]. Due to the critical role of glycolysis in tumorigenesis [13], accumulating data support the idea that tumor suppression may be achieved by inhibiting aerobic glycolysis and promoting oxidative phosphorylation [52]. Although metabolic changes and cancer microenvironments have been the focus of recent attention, the internal mechanism after HCC radiotherapy remains unclear. Despite the report that CTSH increased the chemoresistance of bladder cancer [53], here, for the first time, we have described a CTSH-mediated survival mechanism after IR in HCC cells that substantially contributes to radioresistance. In this context, relatively radioresistant HCC cells were highly addicted to glycolytic metabolism, but when CTSH was knocked down, this cancer metabolic switch was significantly reversed (Figure 2g,h; Figure 3f,g). Here, glucose did not feed the Warburg mainstream aerobic glycolysis to meet the biosynthesis need for cell proliferation, while the cascade of the aerobic respiration cascade was up-regulated (pyruvate intake was also enhanced by MPC1 elevation), which was a strong destruction of the cellular immortality of HCC. With decreased biosynthesis supply and increased oxidative stress brought by aerobic respiration (OXPHOS), the radioresistance was reversed in HCC cells.

Aberrant gene expression is a hallmark of cancer, and cancer cells are always under tremendous pressure to be selected for the pro-survival genetic phenotype [13]. Therefore, our discovery can explain the paradox that CTSH is highly expressed and associated with poor prognosis in certain types of human cancer, such as HCC, but has relatively low expression in other cancers. The high CTSH expression cancers are likely to be highly malignant. In this context, CTSH appears to be important to maintain cancer metabolism in cells with higher glycolytic activity but it is not so important for cancer cells that have less glycolytic activity. Furthermore, the up-regulation of the CTSH protein in HepG2 cells after IR gave us a definitive answer to explain the phenomenon that the HepG2 cell was more resistant to radiation than the MHCC97H cell, in which the CTSH expression did not increase as significantly as in the HepG2 cell (Figure 2e,i,j). From this perspective, we found a novel potential mechanism of CTSH to manipulate HCC metabolism and cell death.

### 3.2. CTSH Knockdown Promotes Apoptosis Following Reversed Metabolic Switch

CTSH is a member of the cathepsin family and functions mostly as an endopeptidase important for the overall degradation of proteins in lysosomes. Despite previous reports that the cathepsin family promotes apoptosis [54,55,56], it has only been reported to protect insulin-secreting and immune β-cells against cytokine-induced apoptosis by perturbing the JNK pathway in type-1 diabetes [57,58]. However, whether CTSH is involved in the cell death of cancer still remained unclear. Here, firstly, we originally found that CTSH knockdown could facilitate the apoptotic process through mitochondrial signaling (such as AIFM1 signaling) in HCC cells. Besides the proapoptotic effect, AIFM1 is also known as an OXPHOS-promoting factor [59]. As OXPHOS promotes the accumulation of oxidative stress and thus the apoptotic cascade [14,15,16], the reversed cancer metabolic switch is related to apoptosis. Secondly, we discovered that CTSH knockdown could facilitate the apoptotic process by inhibiting the activity of the IAP family rather than directly activating the caspase family (although the latter is more widely recognized [60]). Thirdly, a decrease in MMP (IR-induced) and membrane stability (CTSH KD-induced) synergistically combined to promote apoptosis after IR release of the above molecules. In summary, this proapoptotic effect was conducted via not only a normal caspase-dependent pathway but also an independent pathway (AIF signaling) via aerobic respiration enhancement.

### 3.3. CTSH Together with Its Targets Show Potential Value in HCC Treatment

Consistent with these laboratorial results, our clinical bioinformatic analysis also revealed that the CTSH-mediated cancer metabolic switch (Warburg effect) likely contributed to tumorigenesis and was correlated to poor prognosis in clinical HCC cohorts on both a transcriptome and a proteome level. In addition, this mechanism likely acted as a common feature among patients with higher CTSH levels in other cancers. As studies on inhibitors of the cathepsin family have been relatively well developed [61,62], and some of them are even Food and Drug Administration approved for other indications [63,64], they become possible options for clinical application. Therefore, these findings implicate the significant potential of translational medicine for diagnosis and therapy in HCC and other IR-resisting cancer types.

In summary, our study describes an integration of HCC metabolism regulated by CTSH signaling, which mediates radioresistance through promoting glycolysis and inhibiting apoptosis due to the impairment of the release and expression of proapoptotic proteins in HCC cells. With the gradual blossoming of RT in HCC treatment, understanding such tumor metabolic vulnerability and elaborating the underlying mechanism may help to determine more effective combination therapeutic strategies for patients with HCC.

#### Limitations of the Study

In this study, we elucidated a comprehensive CTSH-involved cancer metabolism that inhibits apoptosis in driving radioresistance. However, the direct CTSH regulatory targets affecting the metabolic and apoptotic processes still need further study. More research will be accomplished in the future. In addition, more experiments in vivo will be conducted to further verify our findings. Prospectively, these works will be meaningful for therapeutic purposes in antagonizing HCC radioresistance.

## 4. Materials and Methods

### 4.1. Reagents and Antibodies

An apoptosis inhibitor ZVAD (HY-16658) was obtained from MCE (Princeton, NJ, USA). Primary antibodies CTSH (sc-398527), FAS (sc-8009), Survivin (sc-17779), XIAP (sc-55550), DIABLO (sc-393118), and HTRA2 (sc-365594) were purchased from Santa Cruz Biotechnology (Dallas, TX, USA); GAPDH (#5174S), AIF (#7495S), CASP9 (#9502), CASP3 (#9662), and TOM20 (42406S) were purchased from Cell Signaling Technology (Danvers, CO, USA); and anti-actin was purchased from Sigma Aldrich (Saint Louis, MO, USA, prod. no. A3853). Secondary antibodies goat antirabbit IgG- (H+L) HRP conjugate (cat. no. 170-6515) and goat anti-mouse IgG- (H+L) HRP conjugate (cat. no. 170-6516) were obtained from Bio-Rad Laboratories (Mississauga, ON, Canada).

### 4.2. Western Blot

Cells were lysed in lysis buffer containing protease inhibitors (aprotinin, leupeptin, and PMSF) on ice, and centrifuged at 10,000× *g* for protein collection. Intestinal protein extracts were separated by 12% SDS-PAGE and transferred to nitrocellulose membranes using the Protean Mini Cell (Bio-Rad). After completion of the transfer, membranes were blocked with 5% nonfat milk in TBS/0.1% Tween 20 for 120 min. Incubation with the primary antibody (as indicated) was conducted overnight at 4 °C. Incubation with a peroxidase conjugated anti-mouse or anti-rabbit secondary antibody (1: 10,000) was performed for 120 min at room temperature. Finally, chemiluminescent analysis was performed.

### 4.3. Cell Lines and Cell Culture

Human HCC cell lines HepG2, MHCC97H, and Huh-7 were obtained from the Chinese Academy of Sciences cell bank (Beijing, China). All cells were routinely cultured in Dulbecco’s modified Eagle’s medium (DMEM) (Sigma, Saint Louis, MO, USA) containing 10% fetal bovine serum (Solarbio, Beijing, China) at 37 °C in a humidified atmosphere of 5% CO_2_.

### 4.4. Irradiation

For experiments in vitro, an X-ray generator (X-RAD 320 ix, Precision X-ray Inc., North Branford, CT, USA) was utilized to deliver radiation at a dose rate of 300 cGy/min. The irradiation conditions were as follows: 20 kV, 12.5mA, filter 1, SSD 70 cm; the fractionated dose was 7Gy/9Gy/12Gy × 3 fraction and the single dose was 15 Gy in cells.

For experiments in vivo, an X-ray accelerator (Clinac 23EX, Varian Medical Systems, Inc., Palo Alto, CA, USA) was utilized to deliver radiation at a dose rate of 400 MU/min. The irradiation conditions were as follows: 6MV, distance (SSD) 96.1 cm. For the imitation of the clinical SBRT condition, the dose was 7Gy/9Gy × 3 fraction.

### 4.5. Analysis of Mitochondrial Membrane Potential (MMP)

Mitochondrial membrane potential was detected by a DIOC6 (D273, Invitrogen, USA) probe according to the manufacturer’s instructions. Cells were seeded in p35 plates with a density of 2.5 × 10^4^/mL with 3 parallel wells in each group. After various treatments, cells were stained with a DIOC6 probe at a final concentration of 5 nM at 37 °C in the dark for 25 min. Then, cells were washed with PBS three times and were analyzed using a flow cytometer (ACEA NovoCyte 2040R, ACEA Biosciences, Inc., San Diego, CA, USA); the percentage of M2-2 was used to represent the membrane potential change and for analysis.

### 4.6. Analysis of Apoptosis by Cytometry

Apoptosis was examined by Annexin V (BD, #556420, Franklin Lakes, NJ, USA) staining according to the manufacturer’s instructions. Cells were seeded in p35 plates with a density of 2.5 × 10^4^/mL with 3 parallel wells in each group. After various treatments, cells were divided and stained with /PI at 37 °C in the dark for 20 min. Then, cells were washed with PBS three times, and FITC (Annexin V apoptosis signal) was detected and analyzed using a flow cytometer (ACEA NovoCyte 2040R, ACEA Biosciences, Inc., San Diego, CA, USA). Quadrants 4-2 and 4-4 were used to represent the apoptosis rate.

### 4.7. Lentiviral Production

Lentiviral short hairpin RNA (shRNA) vector-targeting CTSH (pLKO.1-shCTSH) was constructed according to the Oxidative Medicine and Cellular Longevity protocol of pLKO.1-blasticidin vector (Addgene, Cambridge, MA, USA). Then, the forward oligo and reverse oligo were annealed and inserted into the pLKO.1-blasticidin vector: 

CTSH shRNA sequence: 

forward oligo:

CCGG—GACGCAAAGATCACCAGCCAT—CTCGAG—ATGGCTGGTGATCTTTGCGTC—TTTTTG;

reverse oligo:

AATTCAAAAA—GACGCAAAGATCACCAGCCAT—CTCGAG—ATGGCTGGTGATCTTTGCGTC.

### 4.8. Transfection

Target fragments were inserted into lentiviral vectors pLKO.1. All plasmids were verified using DNA sequencing. Together with pMD2G and psPAX2 plasmids, recombinant lentiviral plasmids were transfected into HEK293T cells, in which recombinant lentivirus was generated, and then virus-containing supernatant was collected 48 h after transfection. Target cells were incubated in lentivirus supernatant supplemented with 10 μg/mL polybrene (Sigma-Aldrich, H9268, Saint Louis, MO, USA), and then followed by drug selection with 7 μg/ mL Blasticidin for 7 to 14 days. After the efficiency of knockdown was confirmed via Western blot, surviving cells were used for further experiments.

### 4.9. Cell-Death Analysis by Trypan Blue Staining

Trypan blue (Solarbio, Beijing, China) was used according to the manufacturer’s protocol. Cells were seeded in 6-well plates (3 × 103 cells/well) and collected, then analyzed after 15 min of trypan blue incubation using a flow cytometer (NovoCyte 2040R, ACEA Biosciences, Inc., San Diego, CA, USA). M2-2 was used to represent the cell-death portion and for analysis.

### 4.10. Immunofluorescence Co-Localization Assay

A total of 48 h after transfection, cells were separated into plates, and slides were added before being divided. The cells were collected when they had completely adhered to the coverslip, fixed with 4% paraformaldehyde for 20 min, and then subjected to 0.1% TritonX-100 treatment for 15 min. After blocking the cells with 10% normal non-immune goat serum for 1 h, the cells were treated with the appropriate primary antibodies, HTRA2, DIABLO, BIRC2, XIAP, and TOM20, overnight. The next day, cells were incubated with the corresponding secondary fluorescent antibodies for 1 h and then washed with PBS-tween for 5 min × 3 times. The slides were dried at room temperature avoiding light, then antifade solution was added.

### 4.11. Orthotopic Tumor Model Establishment

A rat liver W256 carcinosarcoma tumor-bearing model was used in our preliminary experiment. Because its blood supply characteristics and growth behavior are similar to human liver cancer (mainly supplied by the hepatic artery), this model is widely used in laboratory therapy, imaging diagnosis, invasion and metastasis of liver cancer intervention, and other studies [29,65,66,67,68,69].

Cells collected from ascites were centrifugated and washed with 1 × PBS and then suspended to the required concentration (1 × 107 cells/mL). Six-week-old male Wistar rats weighing 160–180 g were used. After abdominal anesthesia with 5% chloral-hydrate and iodophor disinfection, a 1–1.5 cm vertical incision was made in the left-upper-abdomen below the ribs. Then, the left liver lobe was squeezed out of the incision, and w256 cells were injected into the lobe slowly (about 1.5 × 106 cells in 0.125 mL, using a micro-syringe). Care was taken to ensure that the cells did not flow back out after the needle was pulled out, because this would cause lethal ascites). After resetting the liver and closing the abdomen, the incision was disinfected. Radiotherapy was initiated about 12 days after the establishment.

### 4.12. Bioinformatic Analyses

GSEA was conducted using the GSEA official App. The bioinformatic database analyses conducted in this study are listed here: GEPIA (http://gepia.cancer-pku.cn/, accessed on 18 November 2022); Kaplan-Meier Plotter (https://kmplot.com/analysis/, accessed on 18 November 2022); Protein atlas (https://www.proteinatlas.org/, accessed on 18 November 2022); TNMplot (https://tnmplot.com/analysis/, accessed on 18 November 2022).

### 4.13. Statistical Analyses

Statistical analyses between groups (e.g., Figure 2d,h,j, Figure 4b, and Figure 5c,d) were performed using the *t* test unless otherwise stated. *p* values of * *p* < 0.1, ** *p* < 0.05, *** *p* < 0.01 were considered statistically significant. The unpaired t test was performed to compare between groups during Trypan blue cell death, MMP, and Annexin-V flow-cytometry. The paired *t* test was performed in colony-formation assay analysis. All statistical tests were performed in GraphPad Prism (version 9.0, Boston, MA, USA). The response variables were log-transformed to avoid skewness of the residuals, which also resulted in per-allele effects expressing percentagewise and not absolute changes of the response variable.

## 5. Conclusions

In summary, our study describes the integration of HCC metabolism regulated by CTSH signaling, which mediates radioresistance by promoting glycolysis and inhibiting apoptosis due to the impairment of the release and expression of proapoptotic proteins in HCC cells.

## Data Availability

Data are contained within the article or supplementary material.

## Supplementary Materials

The following supporting information can be downloaded at: https://www.mdpi.com/article/10.3390/ijms24065257/s1.
