# Supplementary material for: Cathepsin H Knockdown Reverses Radioresistance of Hepatocellular Carcinoma via Metabolic Switch Followed by Apoptosis
Source: Int J Mol Sci. 2023 Mar 9;24(6):5257. doi: 10.3390/ijms24065257 (PMC10049059; doi:10.3390/ijms24065257)
Supplement: Supplementary file 1 [file ijms-24-05257-s001.zip › ijms-2259383-supplementary.pptx]

## Slide 1
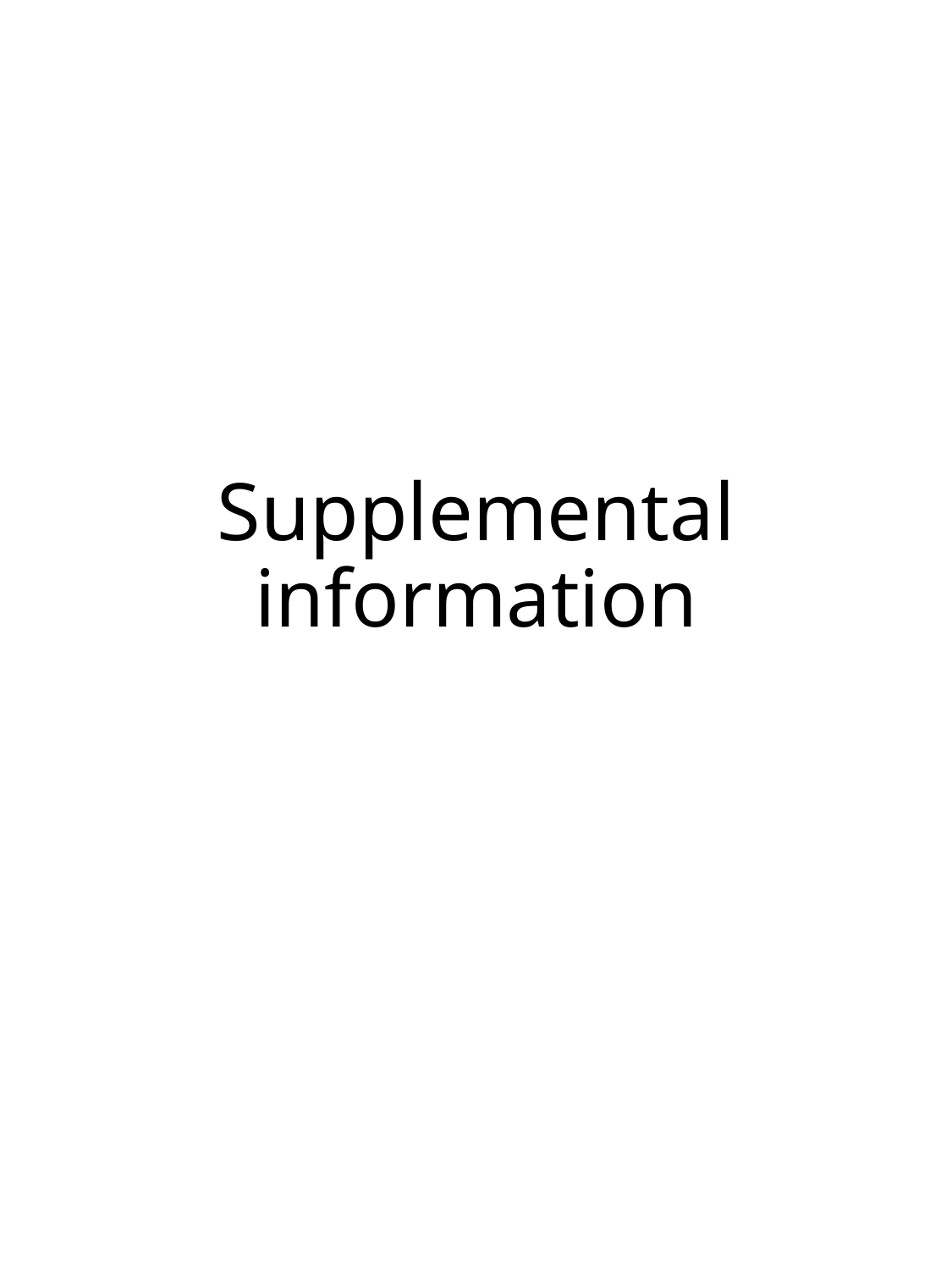

# Supplemental information

## Slide 2
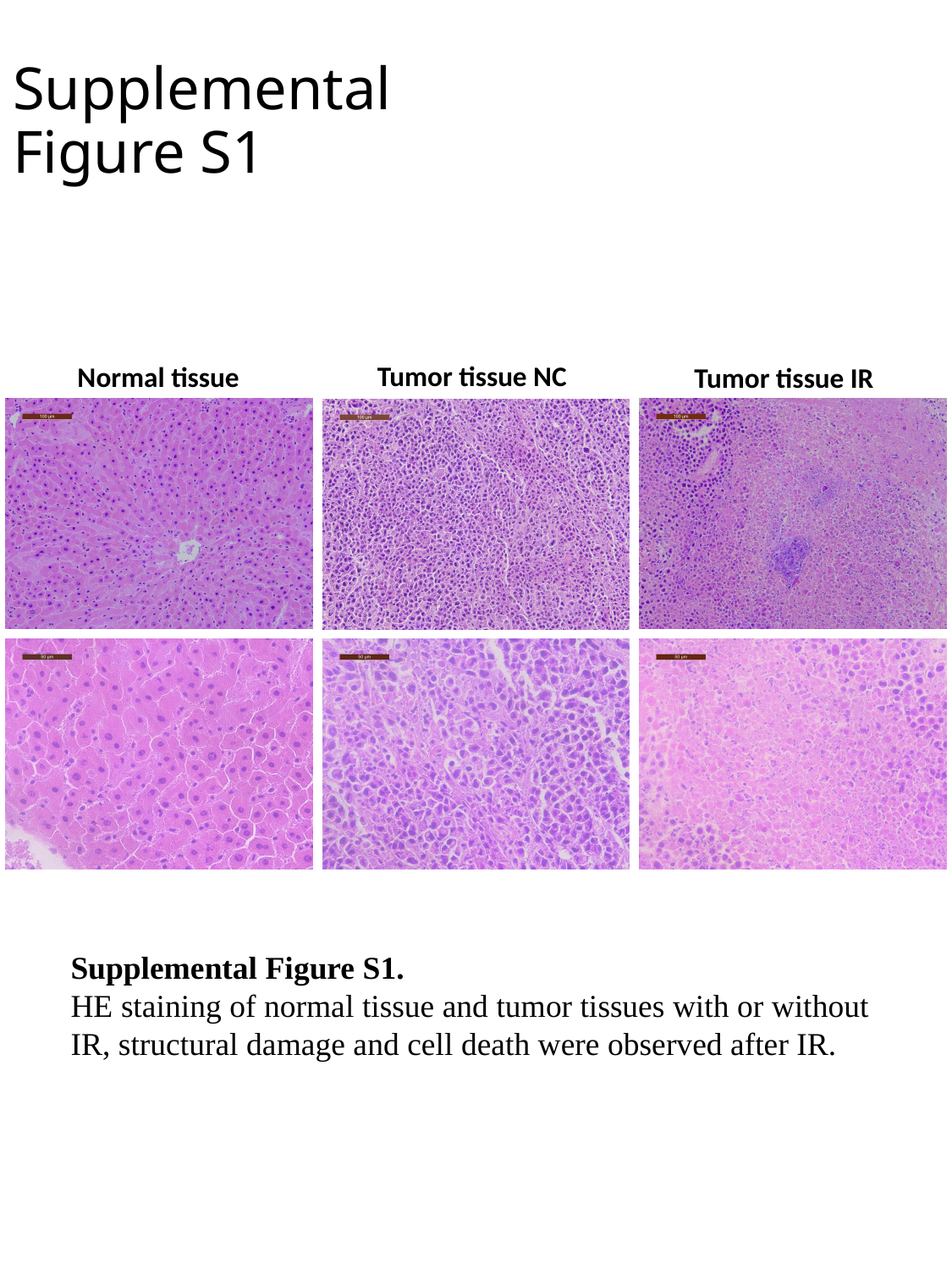

# Supplemental Figure S1
Tumor tissue NC
Normal tissue
Tumor tissue IR
Supplemental Figure S1.
HE staining of normal tissue and tumor tissues with or without IR, structural damage and cell death were observed after IR.

## Slide 3
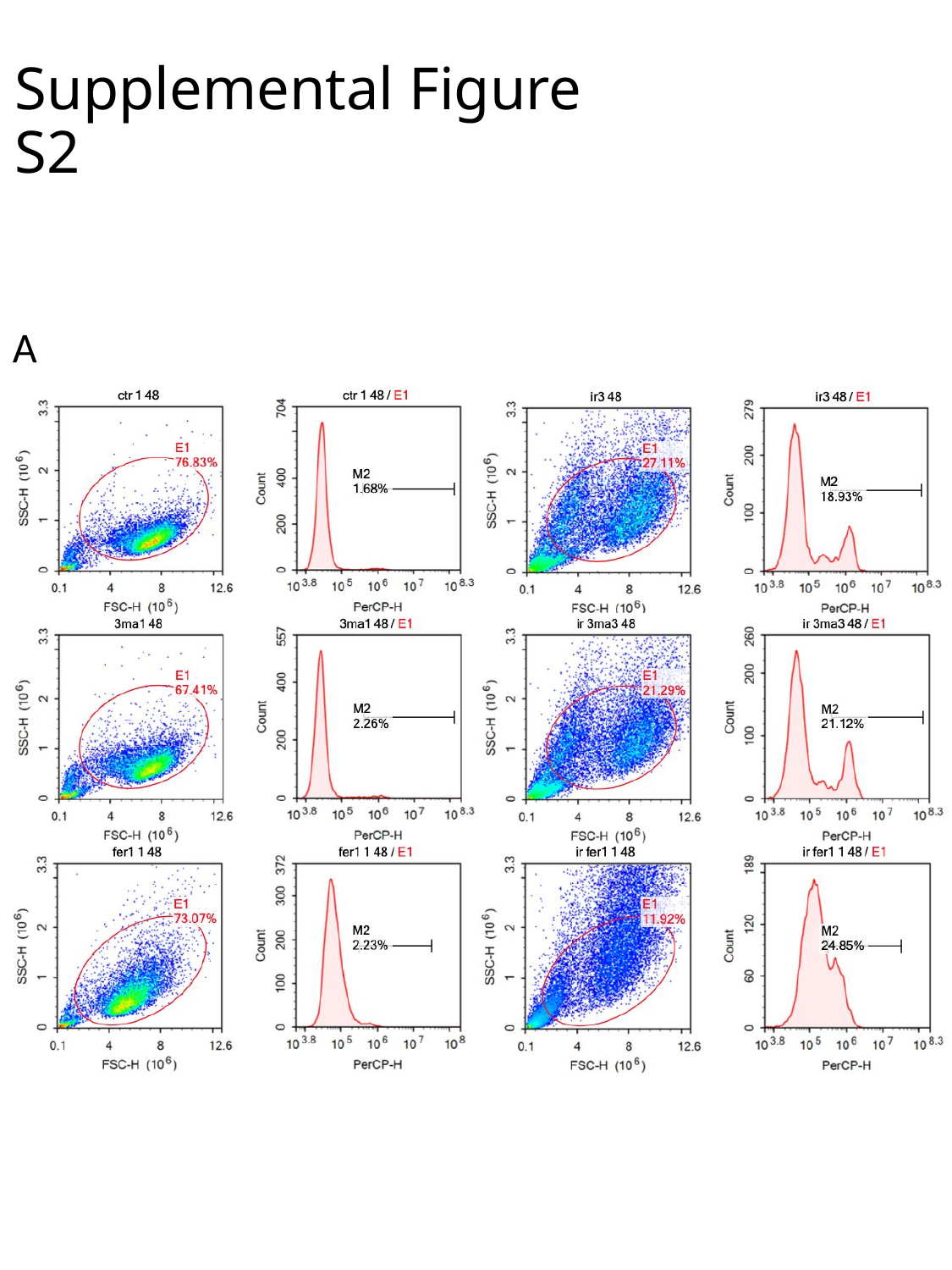

# Supplemental Figure S2
A

## Slide 4
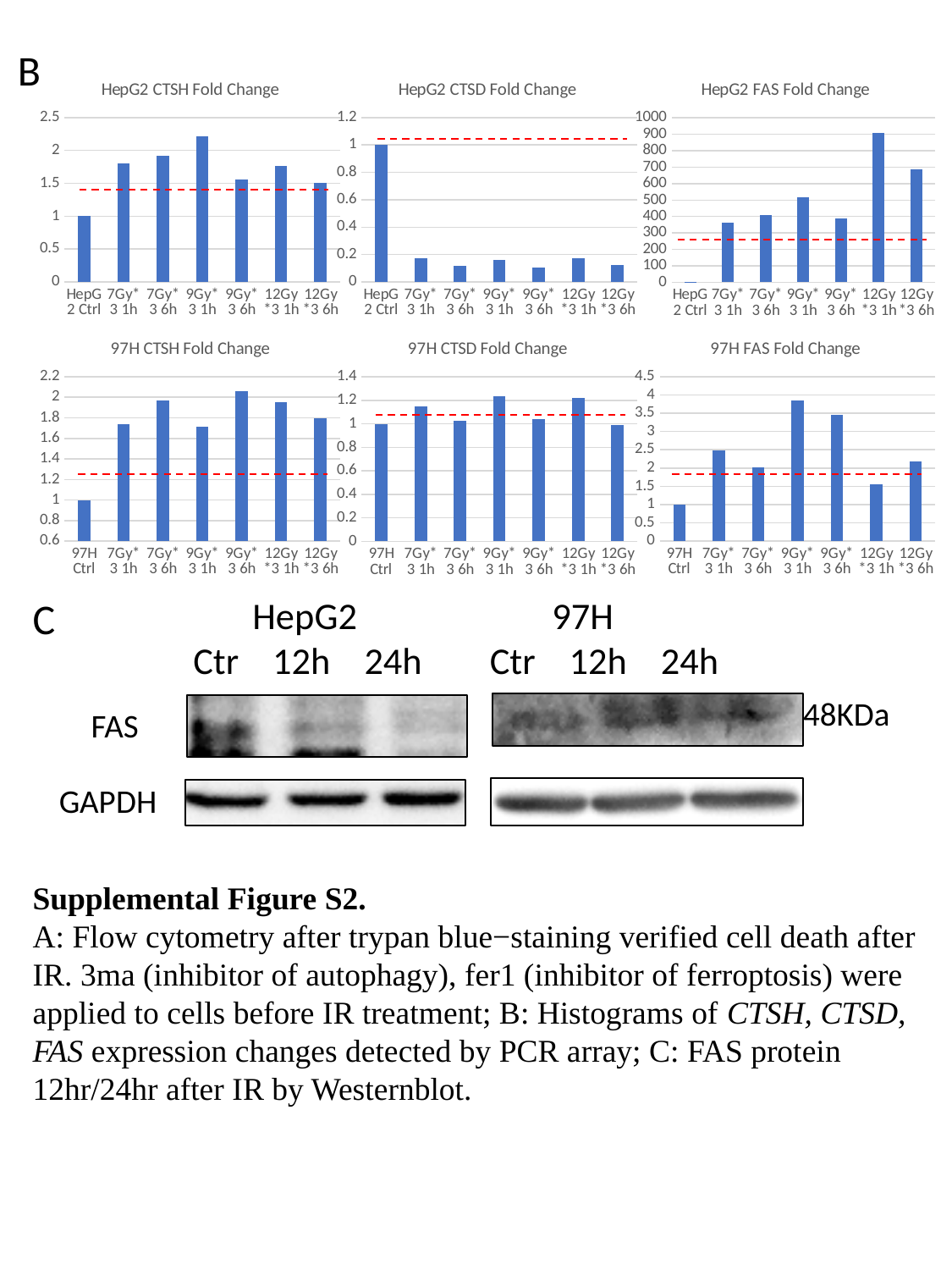

B
### Chart: HepG2 CTSH Fold Change
| Category | CTSH Fold Change |
|---|---|
| HepG2 Ctrl | 1.0 |
| 7Gy*3 1h | 1.79820998731355 |
| 7Gy*3 6h | 1.91580937631817 |
| 9Gy*3 1h | 2.21309415147504 |
| 9Gy*3 6h | 1.5584439531538 |
| 12Gy*3 1h | 1.76764309007648 |
| 12Gy*3 6h | 1.50035941715764 |
### Chart: HepG2 CTSD Fold Change
| Category | CTSD Fold Change |
|---|---|
| HepG2 Ctrl | 1.0 |
| 7Gy*3 1h | 0.1721823812351684 |
| 7Gy*3 6h | 0.11954842579336512 |
| 9Gy*3 1h | 0.16165101974215626 |
| 9Gy*3 6h | 0.10331533166100294 |
| 12Gy*3 1h | 0.17301046347026244 |
| 12Gy*3 6h | 0.1219227157803651 |
### Chart: HepG2 FAS Fold Change
| Category | FAS Fold Change |
|---|---|
| HepG2 Ctrl | 1.0 |
| 7Gy*3 1h | 364.25868992451416 |
| 7Gy*3 6h | 409.28443478485667 |
| 9Gy*3 1h | 517.7121870568452 |
| 9Gy*3 6h | 385.9055435365765 |
| 12Gy*3 1h | 907.322658981759 |
| 12Gy*3 6h | 686.3660559899137 |
### Chart: 97H CTSH Fold Change
| Category | CTSH Fold Change |
|---|---|
| 97H Ctrl | 1.0 |
| 7Gy*3 1h | 1.7383585470468599 |
| 7Gy*3 6h | 1.9683202242895705 |
| 9Gy*3 1h | 1.7127716901877432 |
| 9Gy*3 6h | 2.0612343352491136 |
| 12Gy*3 1h | 1.9538246078334873 |
| 12Gy*3 6h | 1.7992395932159746 |
### Chart: 97H FAS Fold Change
| Category | FAS Fold Change |
|---|---|
| 97H Ctrl | 1.0 |
| 7Gy*3 1h | 2.482927672158258 |
| 7Gy*3 6h | 2.0135733056055227 |
| 9Gy*3 1h | 3.841087872809847 |
| 9Gy*3 6h | 3.4448541474941643 |
| 12Gy*3 1h | 1.5607222573972077 |
| 12Gy*3 6h | 2.1688782677948484 |
### Chart: 97H CTSD Fold Change
| Category | CTSD Fold Change |
|---|---|
| 97H Ctrl | 1.0 |
| 7Gy*3 1h | 1.1497625195032697 |
| 7Gy*3 6h | 1.0239067421559667 |
| 9Gy*3 1h | 1.2317835997828472 |
| 9Gy*3 6h | 1.0440004581133215 |
| 12Gy*3 1h | 1.2198655806845426 |
| 12Gy*3 6h | 0.9890553944364431 | HepG2 97H
 Ctr 12h 24h Ctr 12h 24h
C
48KDa
FAS
GAPDH
Supplemental Figure S2.
A: Flow cytometry after trypan blue−staining verified cell death after IR. 3ma (inhibitor of autophagy), fer1 (inhibitor of ferroptosis) were applied to cells before IR treatment; B: Histograms of CTSH, CTSD, FAS expression changes detected by PCR array; C: FAS protein 12hr/24hr after IR by Westernblot.

## Slide 5
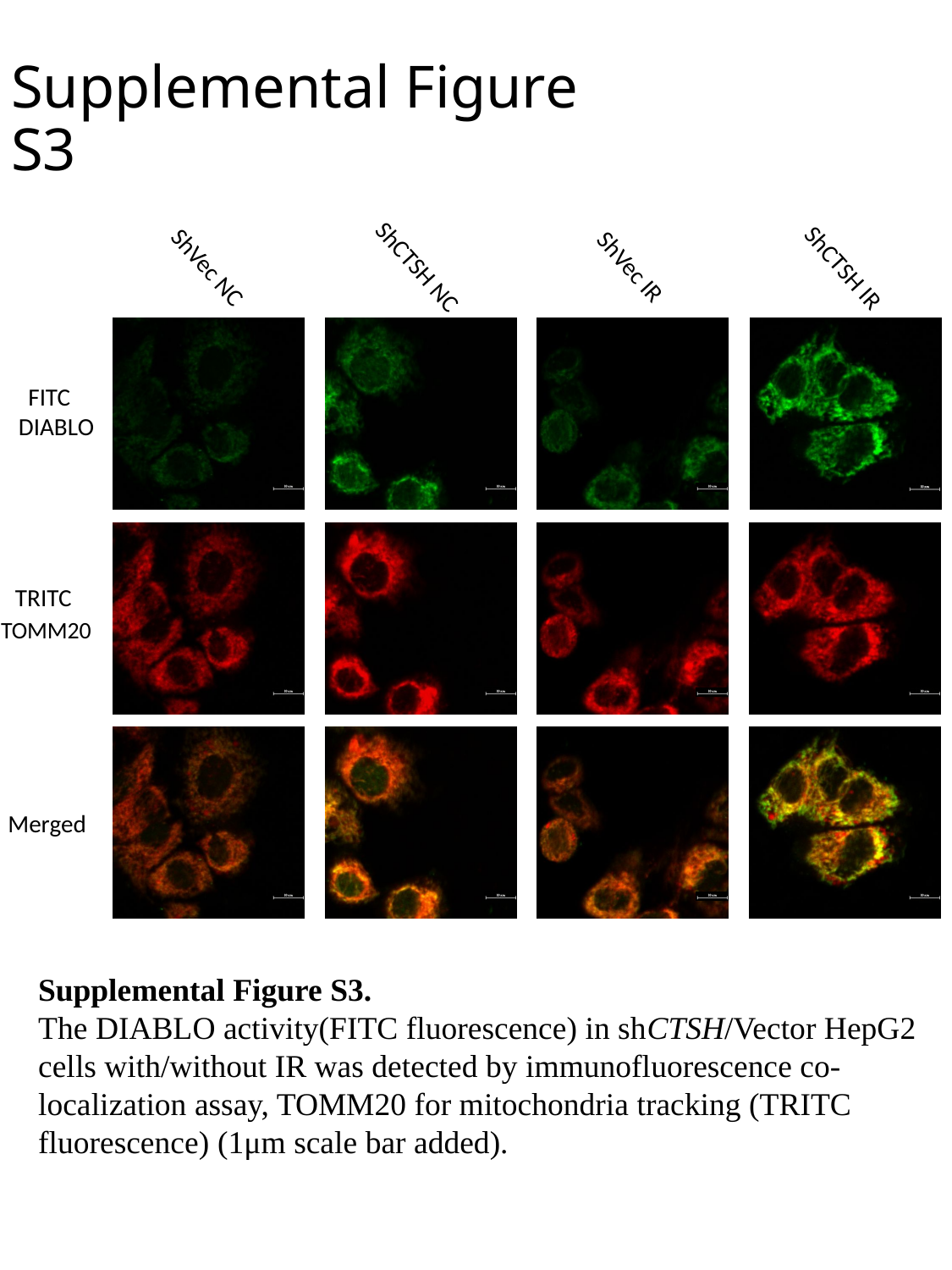

# Supplemental Figure S3
ShCTSH NC
ShVec IR
ShVec NC
ShCTSH IR
FITC
DIABLO
TRITC
TOMM20
Merged
Supplemental Figure S3.
The DIABLO activity(FITC fluorescence) in shCTSH/Vector HepG2 cells with/without IR was detected by immunofluorescence co-localization assay, TOMM20 for mitochondria tracking (TRITC fluorescence) (1μm scale bar added).

## Slide 6
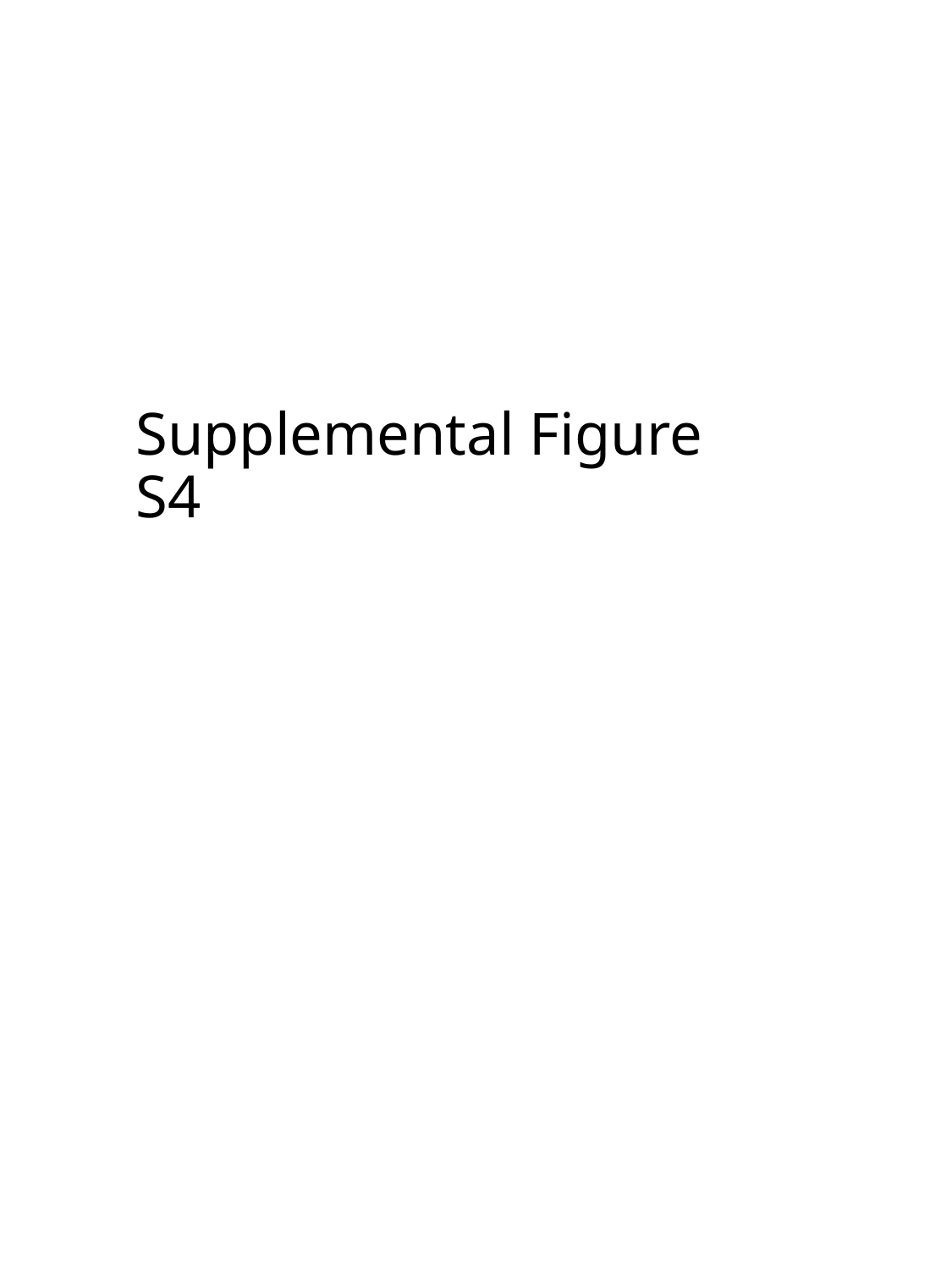

# Supplemental Figure S4

## Slide 7
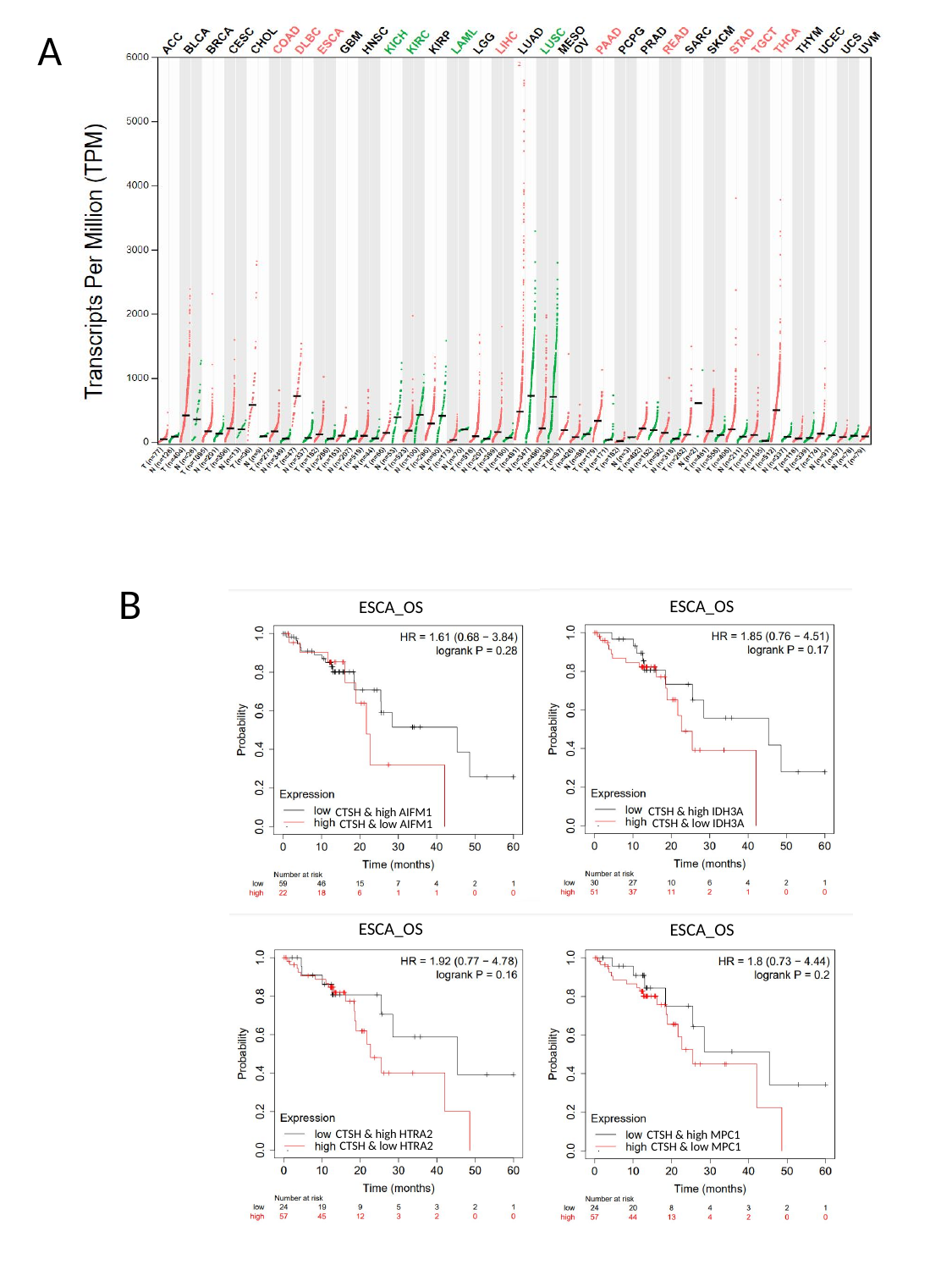

A
B
ESCA_OS
CTSH & high IDH3A
CTSH & low IDH3A
ESCA_OS
CTSH & high AIFM1
CTSH & low AIFM1
ESCA_OS
CTSH & high HTRA2
CTSH & low HTRA2
ESCA_OS
CTSH & high MPC1
CTSH & low MPC1

## Slide 8
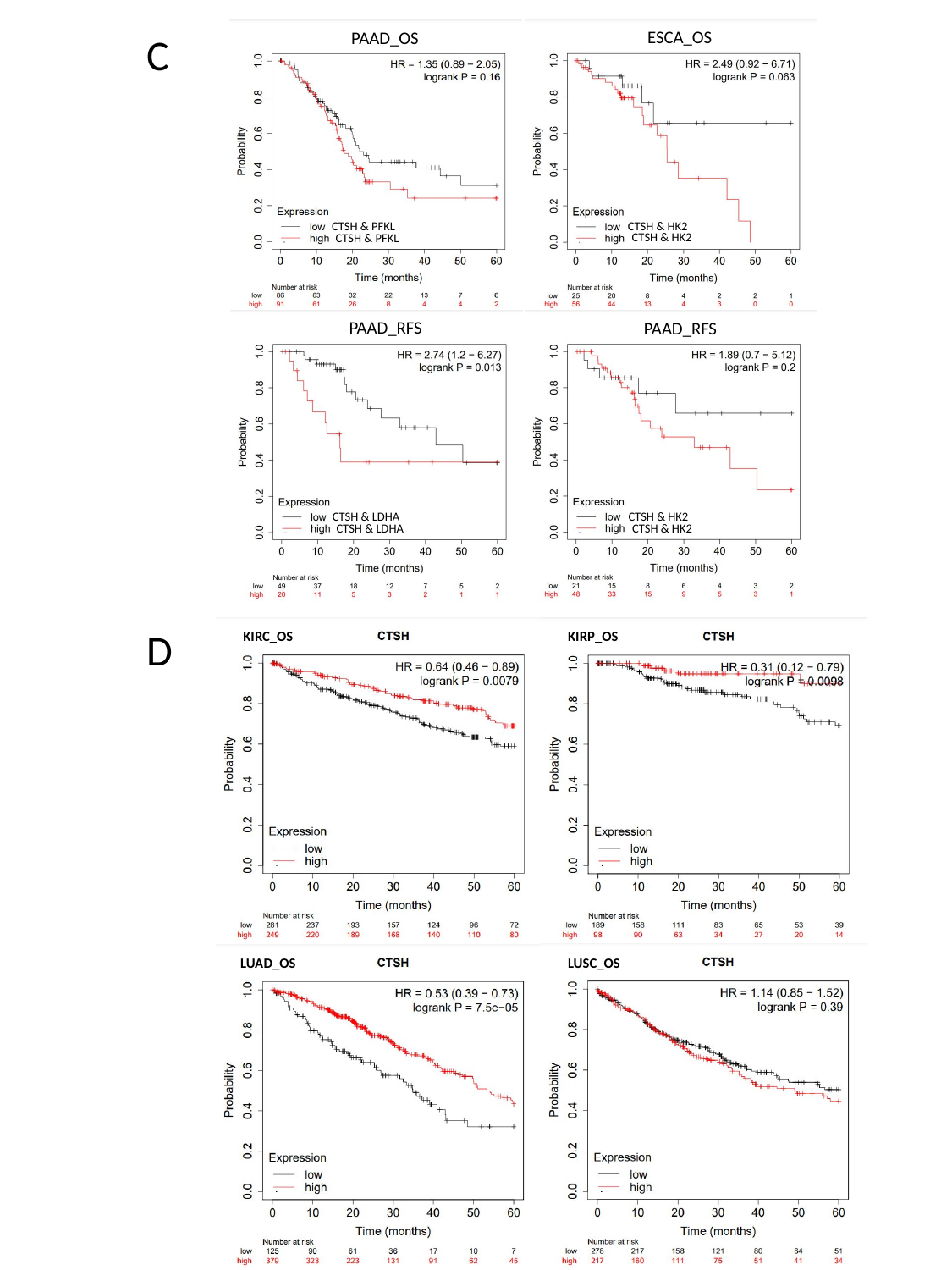

ESCA_OS
CTSH & HK2
CTSH & HK2
PAAD_OS
CTSH & PFKL
CTSH & PFKL
PAAD_RFS
CTSH & LDHA
CTSH & LDHA
PAAD_RFS
CTSH & HK2
CTSH & HK2
C
D
KIRC_OS
KIRP_OS
LUAD_OS
LUSC_OS

## Slide 9
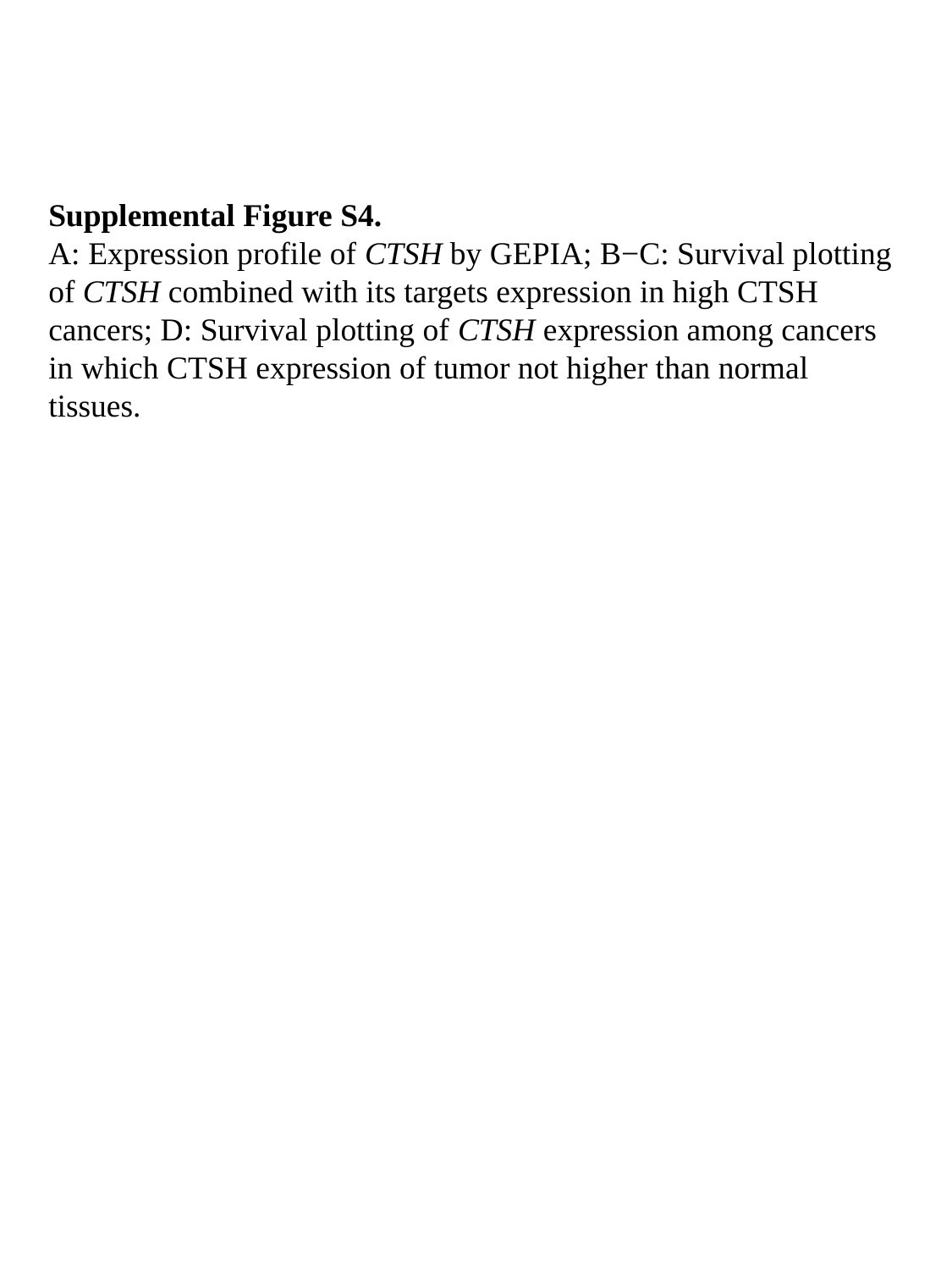

Supplemental Figure S4.
A: Expression profile of CTSH by GEPIA; B−C: Survival plotting of CTSH combined with its targets expression in high CTSH cancers; D: Survival plotting of CTSH expression among cancers in which CTSH expression of tumor not higher than normal tissues.

## Slide 10
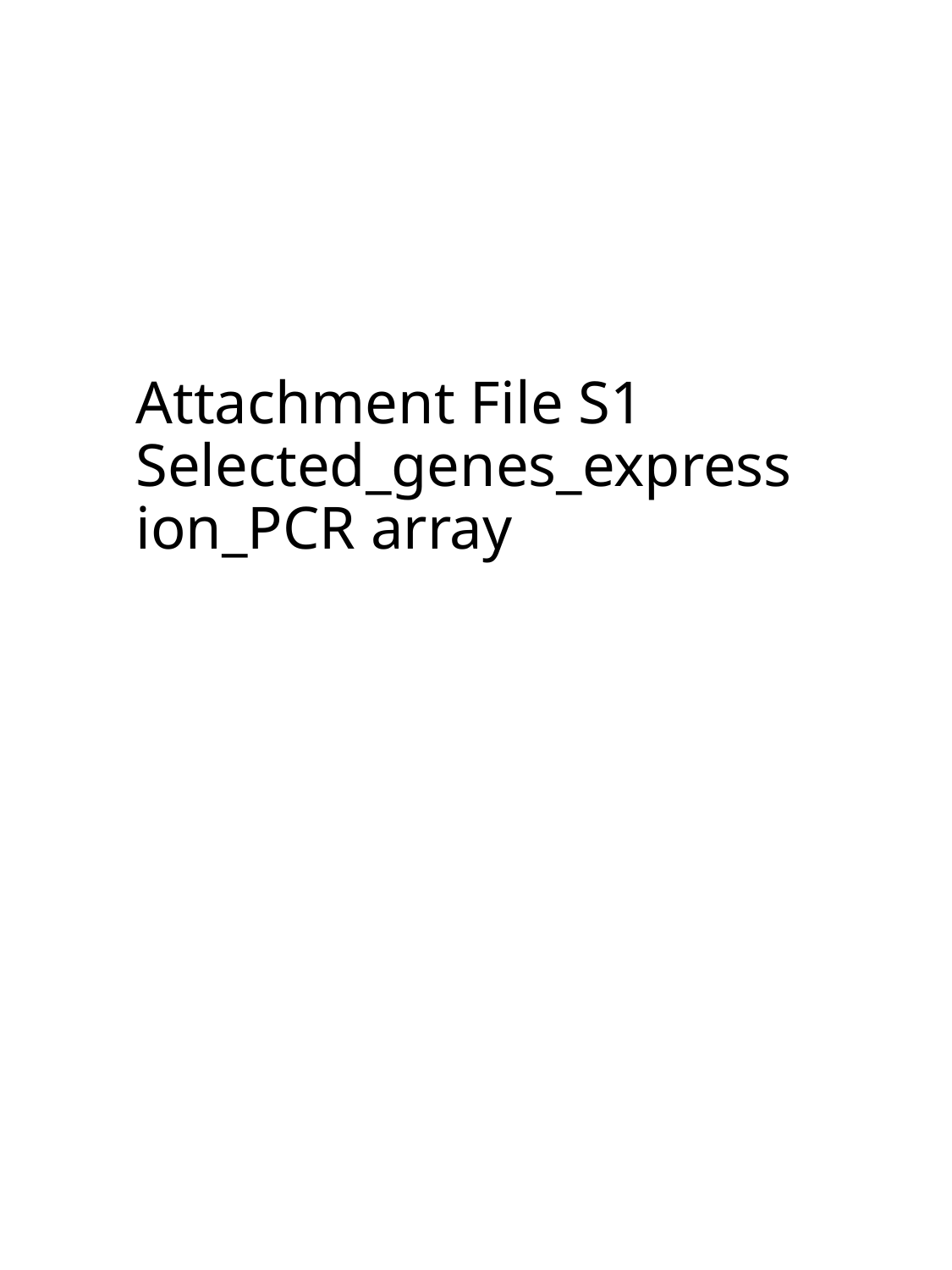

# Attachment File S1 Selected_genes_expression_PCR array

## Slide 11
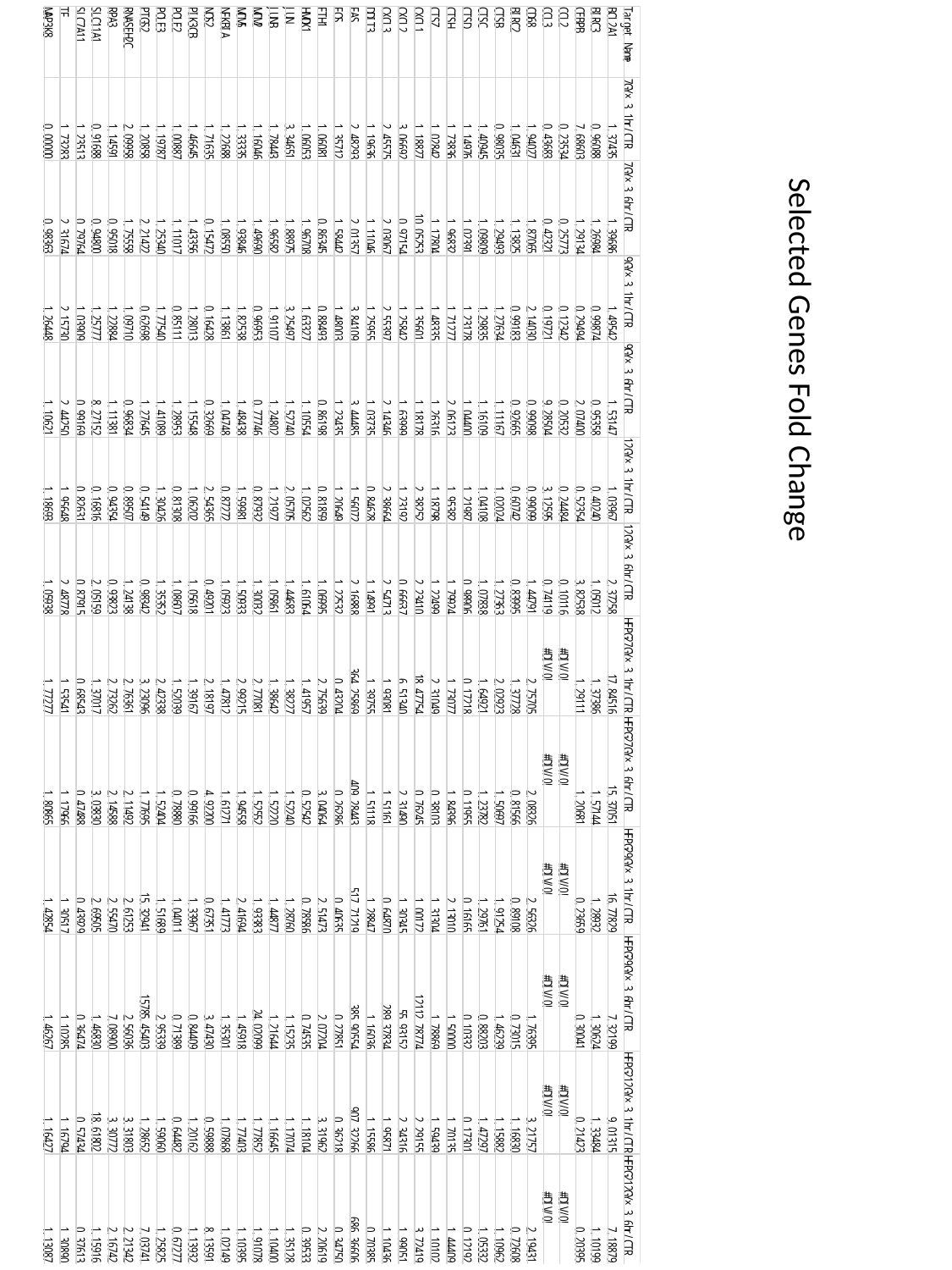

Selected Genes Fold Change

## Slide 12
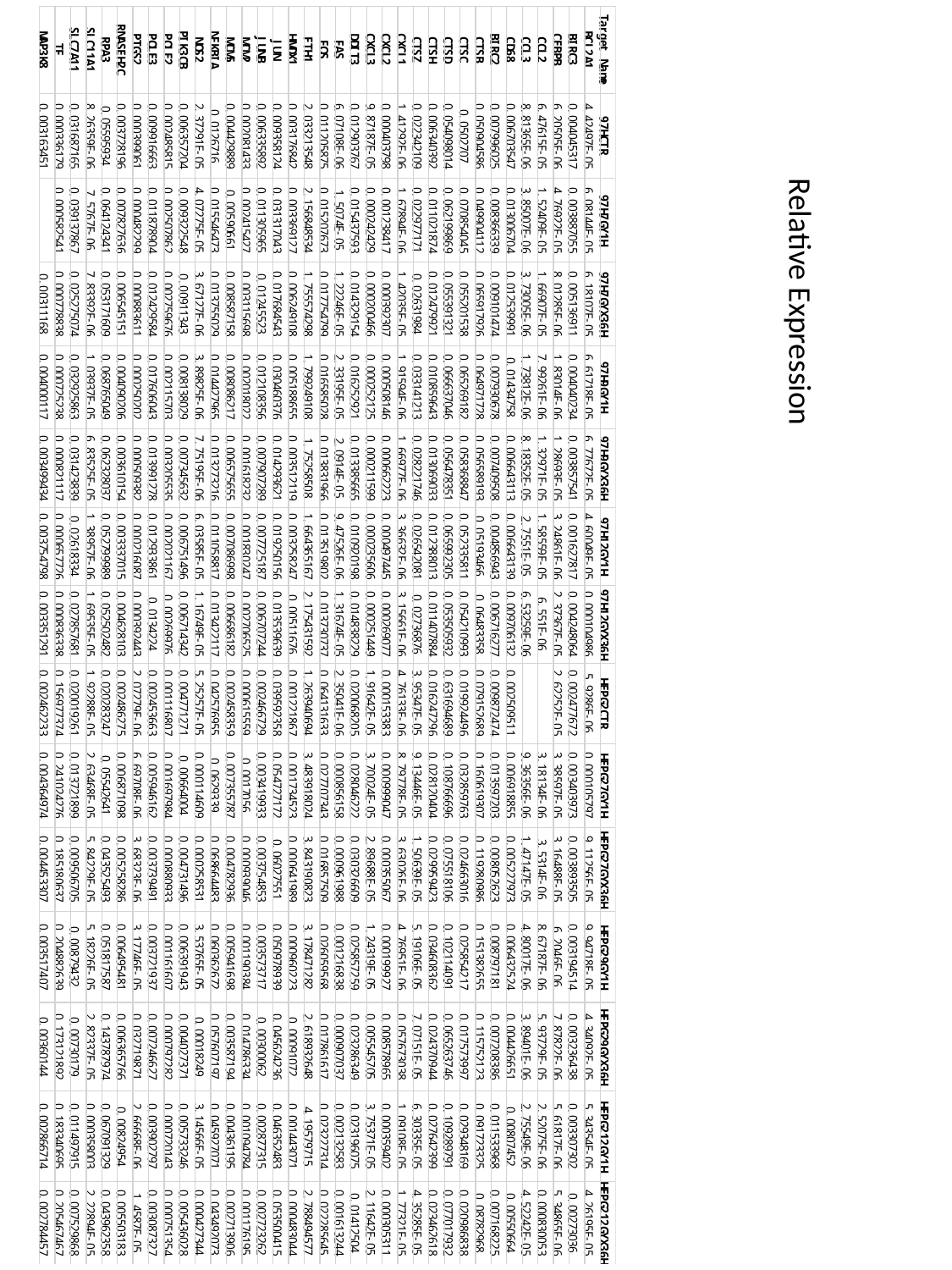

Relative Expression

## Slide 13
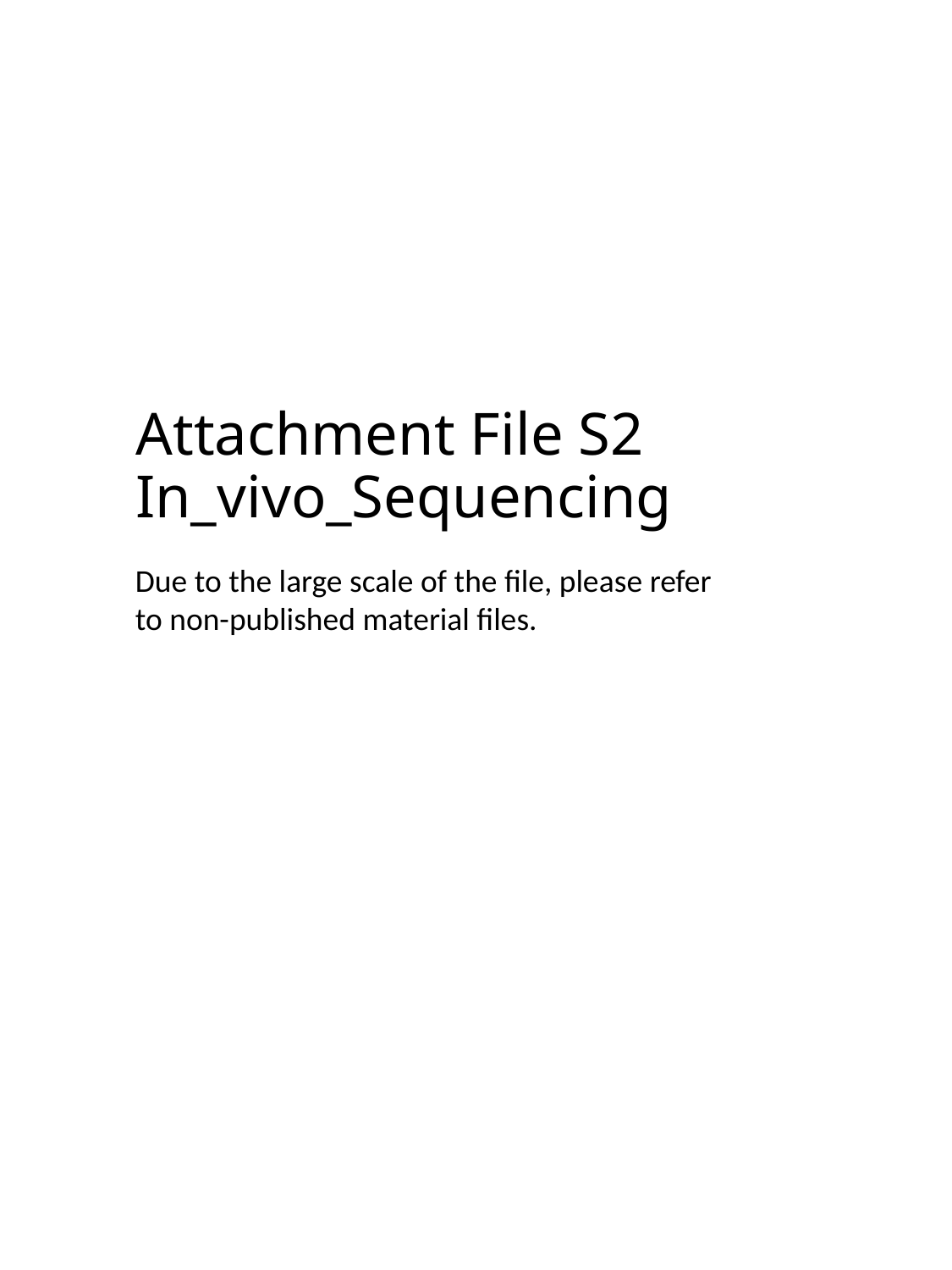

# Attachment File S2 In_vivo_Sequencing
Due to the large scale of the file, please refer to non-published material files.
